# Carboplatin dose calculations for patients with lung cancer: significant dose differences found depending on dosing equation choice

**DOI:** 10.1186/s12885-022-09885-7

**Published:** 2022-07-30

**Authors:** Seçkin Akgül, Bryan A. Chan, Peter M. Manders

**Affiliations:** 1 School of Medicine and Dentistry, Griffith University, Gold Coast, 4215 QLD Australia; 2 Adem Crosby Cancer Centre, Sunshine Coast University Hospital, Birtinya, 4575 QLD Australia

**Keywords:** Lung cancer, Carboplatin, Drug toxicity, Kidney function, eGFR, Cockcroft-Gault, MDRD, CKD-EPI

## Abstract

**Background:**

Carboplatin is the backbone cytotoxic agent for many chemotherapy regimens for lung cancer. Dosing of carboplatin is complicated due to its relationship to renal function and narrow therapeutic index. Overestimation of renal function may lead to supratherapeutic dosing and toxicity, while underestimation may lead to underdosing and therapeutic failure. Although the Modification of Diet in Renal Disease (MDRD) and the Chronic Kidney Disease Epidemiology Collaboration (CKD-EPI) equations have higher accuracy in estimating glomerular filtration rate (eGFR), the Cockcroft Gault (CG) formula has been historically used for carboplatin dosing internationally.

**Methods:**

We compared these formulae to identify patient profiles that were associated with significant carboplatin dose variation by retrospectively analysing the carboplatin dosing of 96 patients with lung cancer. Carboplatin doses were calculated using eGFR generated by MDRD, CKD-EPI ^2009^ and CKD-EPI ^2021^ equations. These three hypothetical doses were compared to actual CG-based doses prescribed.

**Results:**

MDRD and CKD-EPI equations resulted in comparable carboplatin doses; however, CG doses diverged markedly with up to 17% of the patients receiving a carboplatin dose that was at least 20% higher than a non-CG formula would have predicted, and 20% received a dose that was at least 20% lower than a non-CG formula would have predicted. Our data suggest CG use overestimates kidney function in patients with a higher bodyweight and body surface area (BSA) while underestimating it in patients with a lower bodyweight and BSA. Importantly, we demonstrate potential real-world benefit as CKD-EPI predicted lower doses for patients whose (CG-derived) carboplatin dose was later reduced following clinical assessment prior to infusion.

**Conclusions:**

We have therefore confirmed significant differences in carboplatin dosing depending on the equation used in our modern patient population and suggest that use of CKD-EPI provides the most clinically appropriate carboplatin dosing and should be implemented as the new standard of care internationally.

**Supplementary Information:**

The online version contains supplementary material available at 10.1186/s12885-022-09885-7.

## Background

Lung cancer is one of the most common cancers in terms of its global incidence (2.2 million new cases in 2020) and mortality (1.8 million deaths in 2020) [1]. The 5-year survival rate varies between 4–17% depending on the stage and cancer subtype [2]. Although the incidence of lung cancer is decreasing as a result of smoking cessation initiatives, overall lung cancer survival remains low, and mortality is the highest among all cancer types in most parts of the world, emphasizing the ongoing importance of effective systemic treatment strategies [3–5].

Lung cancer is traditionally classified into small-cell lung cancer (SCLC) (~ 15%), and non-small-cell lung cancer (NSCLC) (~ 85%) [6]. Patients with SCLCs typically present with early-onset dissemination and extensive disease at diagnosis in up to 80% of patients [7, 8]. Accordingly, SCLC has the poorest outcome among all lung cancer subtypes with a 5-year survival rate of ~ 5% [9]. Therapeutic improvements are limited to enhanced radiation strategies without any significant breakthrough therapies in the last four decades [2, 10, 11]. This has resulted in reliance on limited cytotoxic chemotherapies and in particular platinum-based alkylating agents (e.g., cisplatin or carboplatin) as the backbone chemotherapy for both limited and extensive stage disease [9, 10, 12, 13]. Similarly, international guidelines recommend cytotoxic chemotherapy, particularly platinum agents, as the standard of care for first-line therapy of advanced NSCLC in conjunction with targeted therapy and immunotherapy [2, 12, 14].

Higher doses or increased exposure to carboplatin increases the risk of myelosuppression (i.e., neutropenia, anaemia, and thrombocytopenia) and hepatotoxicity [15, 16], leading to dose delays, dose reductions, or early discontinuation of chemotherapy [15]. The Renal Insufficiency and Anticancer Medications (IRMA) and the Belgian Renal Insufficiency and Anticancer Medications (BIRMA) studies showed that 50% of the patients with cancer had reduced kidney function, 12–20% had chronic kidney disease (CKD), and 80% received potentially nephrotoxic anticancer drugs [17, 18]. Approximately 71% of the administered carboplatin is excreted unchanged in the urine within 24 h, and another 3–5% is excreted within the following 72 h, suggesting that there is a close relationship between carboplatin exposure and kidney function [16, 19]. Thus, accurate estimation of kidney function has clinical significance, as falsely low estimates can lead to insufficient drug dosing and treatment failure while falsely high estimates can lead to supratherapeutic dosing and toxicity [20, 21].

Carboplatin dose is calculated based on the renal function of each patient in order to minimize the toxicity while increasing the therapeutic efficacy [22]. Glomerular filtration rate (GFR) is used as a surrogate indicator for renal function, and it is estimated (eGFR) by a formula that uses several parameters such as patients’ serum creatinine (SrCr) and/or cystatin C levels, age, weight, sex, and race [23]. The majority of patients with lung cancer are over the age of 65 years and estimation of kidney function is particularly important for the treatment of older patients, as they are more likely to have renal impairment, including CKD [22]. Several equations have been developed to calculate eGFR based on creatinine clearance with the Cockcroft-Gault (CG) being the most widely used formula adopted globally [24]. CG-based eGFR is routinely substituted for GFR in the Calvert equation in the calculation of carboplatin dosage [25]. However, CG fails to sufficiently compensate for non-GFR determinants of SrCr and has significant bias in a real-world population due largely to its original population consisting of only 249 White patients, 96% of whom were men [21, 24].

The revised version of Modification of Diet in Renal Disease (MDRD) study equation with four variables (age, sex, ethnicity, and SrCr levels) was developed to simplify its clinical use, particularly for patients with CKD [23]. However, since this equation was developed by data from people with CKD, it is prone to imprecision and underestimation of kidney function in GFR levels higher than 60 ml/min per 1.73 m^2^ [20, 26]. Most recently, the Chronic Kidney Disease Epidemiology Collaboration (CKD-EPI) equation has been endorsed as the most accurate in estimating GFR [20, 27, 28]. The CKD-EPI equation has been updated over the years with the latest versions, 2021 CKD-EPI Creatinine and 2021 CKD-EPI Creatinine-Cystatin C, which omit the race coefficient [29].

Therefore, we designed a retrospective study to validate the differences between eGFR formulae using data from patients with lung cancer. We then quantified and demonstrated the impact of utilising different formulae on carboplatin dosages and identified the patient characteristics that are most susceptible to this impact. Finally, we established a correlation between clinical dose adjustments and hypothetical dosages predicted by different eGFR formulae to establish the utility of a non-CG formula in clinical oncology. Our goal is to provide representative evidence and illustrate the outcome of different eGFR formulae on carboplatin dosing to ensure the safety of our patients while maintaining the highest achievable therapeutic effect.

## Materials and methods

### Data collection

The current study involves data from 96 patients with lung cancer treated at the Sunshine Coast Hospital and Health Service (SCHHS), QLD, Australia between May 1^st^ 2019 and May 1^st^ 2020. All relevant data were retrieved from the CHARM software, which is an Oncology Information Management Solution. The retrospective data collection commenced in May 2020. The unprocessed data included patient demographics, lung cancer subtype, treatment pathway, carboplatin dosing weight, dosing height, dosing body surface area (BSA), dosing estimated glomerular filtration rate (eGFR), carboplatin administration date, area under the curve (AUC) protocol, carboplatin dose, adjusted dose, and the total number of carboplatin cycle. During analyses, all patients were deidentified with patient ID numbers between 01–96. Patients were predominantly White with a smaller group of mixed races. No Black patient was identified.

### Patient categorisation

Depending on the objective of the analyses, patients were divided into several subgroups, including the lung cancer subtype, AUC protocol, total number of carboplatin cycles, carboplatin dose change based on the employed eGFR formula, and the clinical adjustment of their carboplatin dose. AUC-5 and AUC-6 patients were combined as AUC-5/6, and analysed separately from the AUC-2 patients in the majority of the study.

### eGFR and serum creatinine calculation

Due to clinician variability and local practice, most patients did not have continually updated SrCr, bodyweight, and height values that were measured throughout their carboplatin cycles immediately prior to their drug administration. SrCr was measured by the Jaffe method. The patients received a carboplatin dose based on their initial “dosing eGFR”, which had been calculated by the CG formula using SrCr value and bodyweight measured during prior visits. Therefore, we used these pre-determined dosing values indicated in the treatment charts to calculate the SrCr for each patient for each carboplatin cycle. The formula to conversely calculate the SrCr from eGFR was:

$$\begin{array}{c}SrCr=\frac{\left(140-age\;at\;treatment\right)\times\left(dosing\;weight\right)\times\left(CG\;sex\;coefficient\right)}{\left(72\times dosingeGFR\right)}\\\rangle\;Age\;at\;treatment\;in\;years;dosing\;weight\;in\;kgs;CG\;sex\;coefficient:0.85\;\left(females\right),1.00\;\left(males\right);eGFR\;in\;mL/min.\end{array}$$

**Fig. 1:**
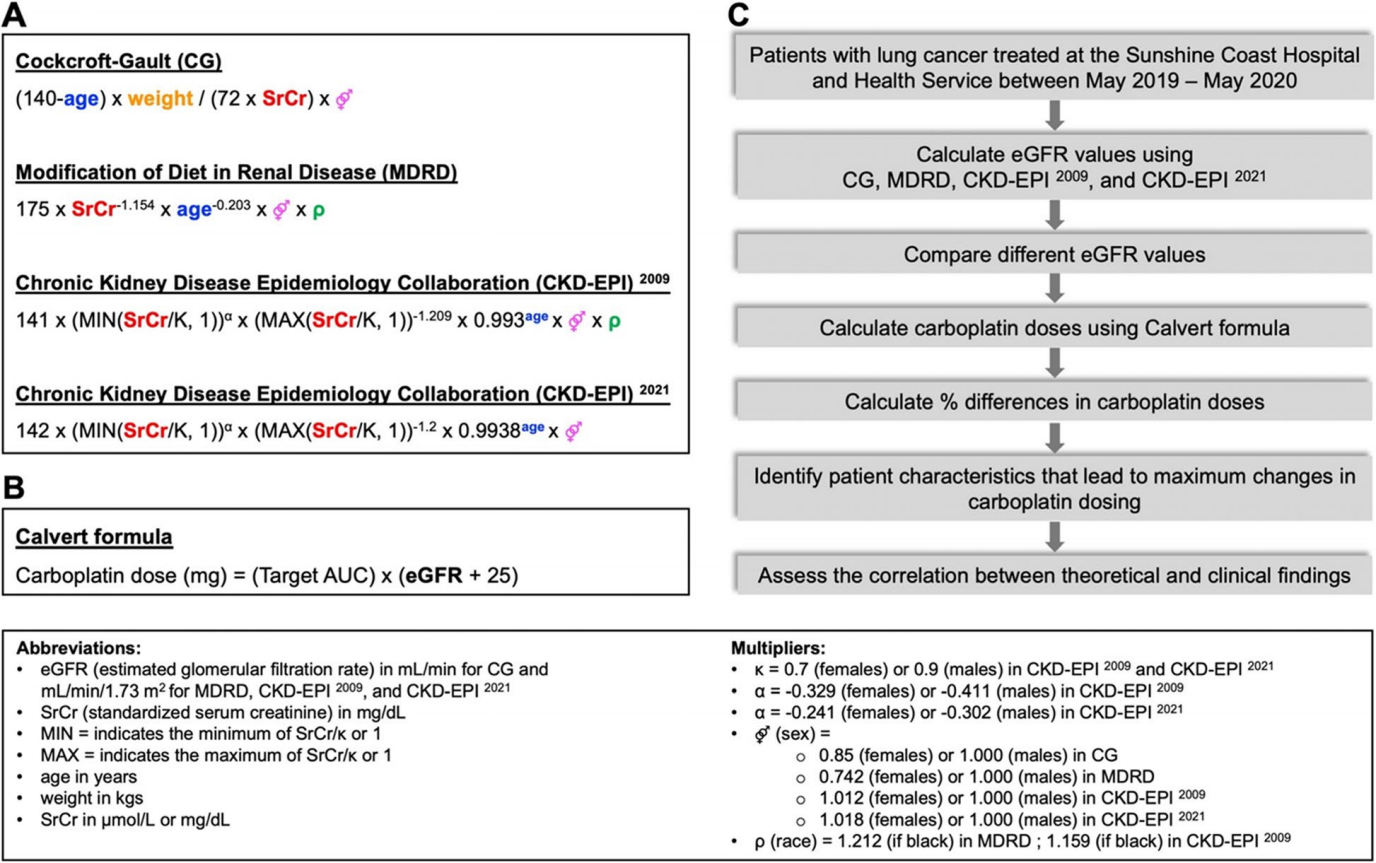
The estimation of the kidney function and calculation of the carboplatin doses in the current study.** A **Cockcroft-Gault (CG), Modification of Diet in Renal Disease (MDRD), Chronic Kidney Disease Epidemiology Collaboration (CKD-EPI) ^2009^ and CKD-EPI ^2021^ are the most common formulae to calculate eGFR. Baseline patient characteristics, including patient age, bodyweight, sex, race, and SrCr, have different emphasis within each formula, resulting in different eGFR values for the same patient. **B **Once eGFR is calculated based on one of the equations, the Calvert formula is used to determine the appropriate carboplatin dose. **C **The research strategy is outlined

This value was then multiplied by 88.4016973 to convert the unit of SrCr from mg/dL to µmol/L. Once SrCr values were determined for each carboplatin cycle, eGFR was calculated using CG, MDRD, CKD-EPI ^2009^, and CKD-EPI ^2021^ formulae as described in Fig. 1A.

### Carboplatin dose calculation and comparison

Carboplatin doses were calculated using eGFR values and the Calvert formula, as described in Fig. 1A and B. The carboplatin doses calculated based on the CG formula were considered as the “original” or “standard” doses, as prescribed in patients’ treatment charts. The dosages calculated based on MDRD, CKD-EPI ^2009^, and CKD-EPI ^2021^ were considered as “hypothetical”, as they were not used during the treatment of the patients in this study. The hypothetical dosages were then compared to the original dose by calculating the percent difference, as described in Results Sect. 4.4. BSA-adjusted carboplatin doses were calculated by multiplying MDRD-, CKD-EPI^2009^-, and CKD-EPI^2021^-derived eGFR values by BSA/1.73. These BSA-adjusted eGFR values were then used in the Calvert formula as described above.

### Statistical analyses

Prism 9 and Microsoft Excel software was used for all analyses. Fisher’s exact test and unpaired, two-tailed t-test were used for statistical comparisons, as indicated in each figure legend. Simple linear regression and goodness of fit (R^2^) was used for determining the relationship between CKD-EPI ^2009^ and CKD-EPI ^2021^. *p* < 0.05 was considered statistically significant.

## Results

### Estimated glomerular filtration rate (eGFR) can be calculated by several formulae

Cockcroft-Gault (CG), Modification of Diet in Renal Disease (MDRD), and Chronic Kidney Disease Epidemiology Collaboration (CKD-EPI) were used to estimate kidney function. The current study employs two versions of the CKD formulae, CKD-EPI ^2009^ and CKD-EPI ^2021^. These four formulae have different emphasis on baseline patient characteristics, including patient age, bodyweight, sex, race, and serum creatinine (SrCr) value, resulting in different eGFR values for the same patient (Fig. 1A).

- 〉 CG formula is the only one that incorporates the bodyweight into consideration, and it is directly proportional to eGFR (i.e., the higher the bodyweight, the higher the eGFR).
- 〉 Female sex is inversely proportional to eGFR in CG and MDRD, but directly proportional to eGFR in CKD-EPI ^2009^ and CKD-EPI ^2021^. However, Κ and α coefficients in CKD-EPI formulae result in a lower eGFR for female patients with SrCr ≤ 0.7 mg/dL (61.89 µmol/L).
- 〉 SrCr is inversely proportional to eGFR in all the formulae (i.e., the higher the SrCr, the lower the eGFR).
- 〉 While older age results in a lower eGFR in CG, CKD-EPI ^2009^, and CKD-EPI ^2021^, it leads to a higher eGFR in the MDRD formula.
- 〉 Finally, MDRD and CKD-EPI ^2009^ formulae have a race coefficient for Black patients, which is omitted in CKD-EPI ^2021^ (Fig. 1A).

Once eGFR is calculated based on one of the equations, the Calvert formula is used to determine the appropriate carboplatin dose (Fig. 1B).

To determine the impact of these formulae on carboplatin dosing in a real-world population, we used a retrospective model involving 96 patients with lung cancer._._ Based on our outlined research strategy (Fig. 1C), we first calculated eGFR values for all patients using each formula. We then compared these four different eGFR values for each patient and followed a similar approach for assessing hypothetical carboplatin doses calculated based on different eGFR values. Finally, we evaluated the correlation between our theoretical findings and clinical findings.

### Patients with lung cancer are treated with different area under the curve (AUC) target and carboplatin cycle number

**Fig. 2:**
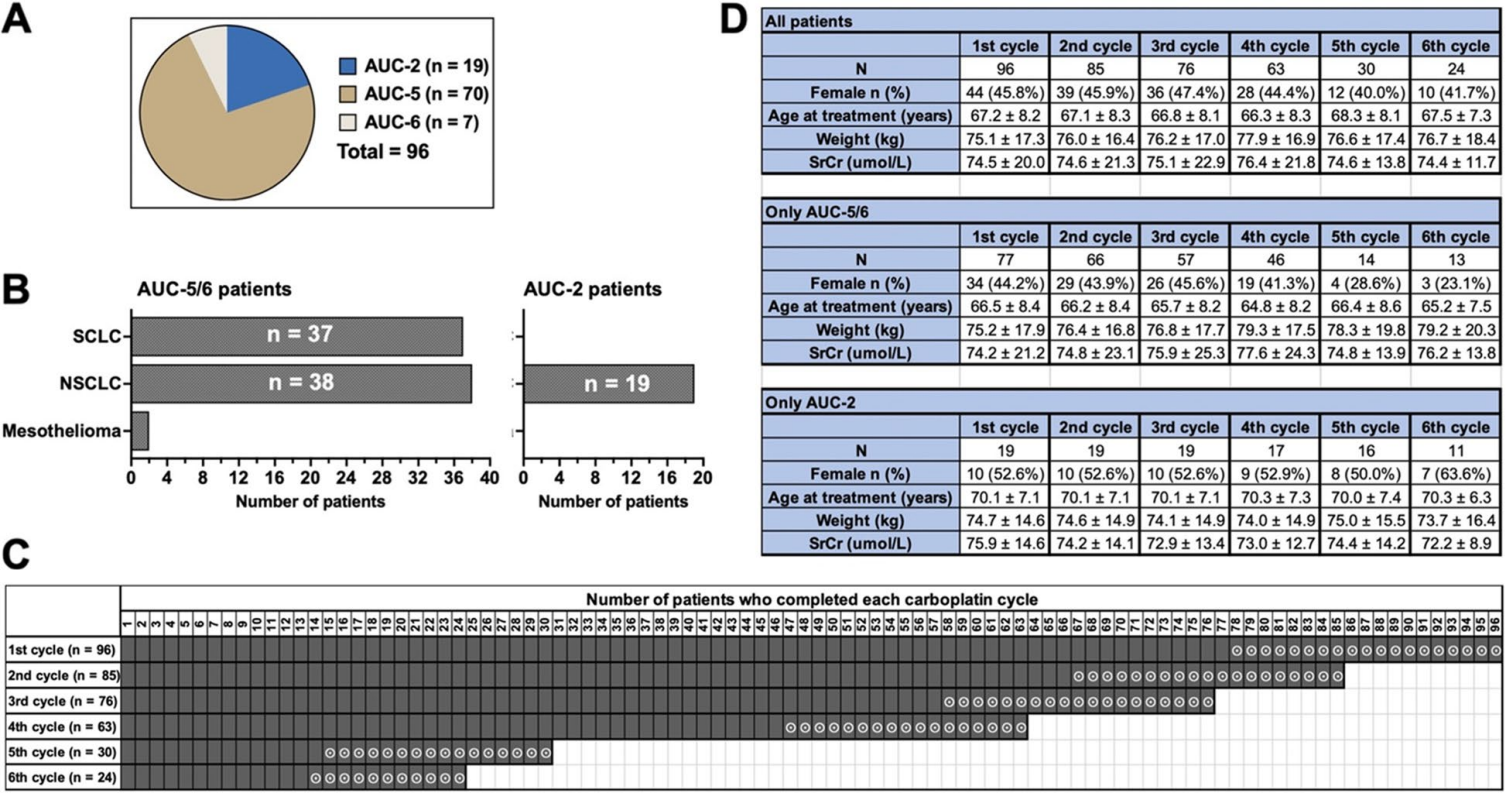
Baseline characteristics and treatment regimens of patients with lung cancer. **A **The distribution of patients based on different carboplatin treatment regimens (i.e., AUC-2, AUC-5, and AUC-6) at the beginning of the treatment protocol. **B** Distribution of lung cancer types among different AUC groups. **C **The number of patients who completed each carboplatin cycle. Boxes with ⨀ indicate AUC-2 patients, others are for AUC-5/6 patients. **D **The baseline patient characteristics, including sex, age at diagnosis, weight, and serum creatinine (SrCr) levels across 6 cycles of carboplatin treatment. The top table includes data from all patients, the middle table from AUC-5 and AUC-6 patients (i.e., AUC5/6), and the bottom table from AUC-2 patients (i.e., AUC-2). Values are presented as mean ± standard deviation

Of the 96 patients with lung cancer, the majority (73%) were treated using an AUC-5 protocol, and the remainder were treated with an AUC-2 (20%) or AUC-6 (7%) protocol (Fig. 2A). Patients with small cell lung cancer (SCLC) made up 39% of the cohort and were treated based on either AUC-5 or AUC-6 (AUC-5/6, hereafter) (Fig. 2B). 59% of the patients were diagnosed with non-small cell lung cancer (NSCLC), two-thirds of whom were treated with AUC-5/6, and the remaining third was treated with AUC-2. An AUC-2 protocol was used exclusively in patients with NSCLC undergoing concurrent radiotherapy. There were two patients with mesothelioma, and both patients were treated based with AUC-5 (Fig. 2B).

Patients were scheduled for four or six cycles of chemotherapy depending on clinical assessment. We analysed the number of patients who completed each carboplatin cycle (Fig. 2C). There was a relationship of decreasing patient participation due to toxicity or other clinical complications over time. A total of 96 patients were commenced on carboplatin treatment, and approximately 66% of them completed four cycles, while only 25% received six cycles (Fig. 2C and D).

We then investigated whether there was an overrepresentation of patient characteristics in any of the carboplatin cycles. However, none of the variables of sex, age at treatment, bodyweight, or SrCr value were significantly different when compared across the six carboplatin cycles (Fig. 2D). Approximately 42–46% of the patients were female, age at treatment was 66–68 years, bodyweight was 75–78 kg, and SrCr value was 74–76 μmol/L. Except for the third and fourth cycles where AUC-2 patients were significantly older than the AUC-5/6 patients, there was not any significant difference between AUC protocols based on these patient characteristics (Fig. 1D).

### CKD-EPI equations result in a narrower spectrum of eGFR values and carboplatin doses than CG and MDRD

**Fig. 3:**
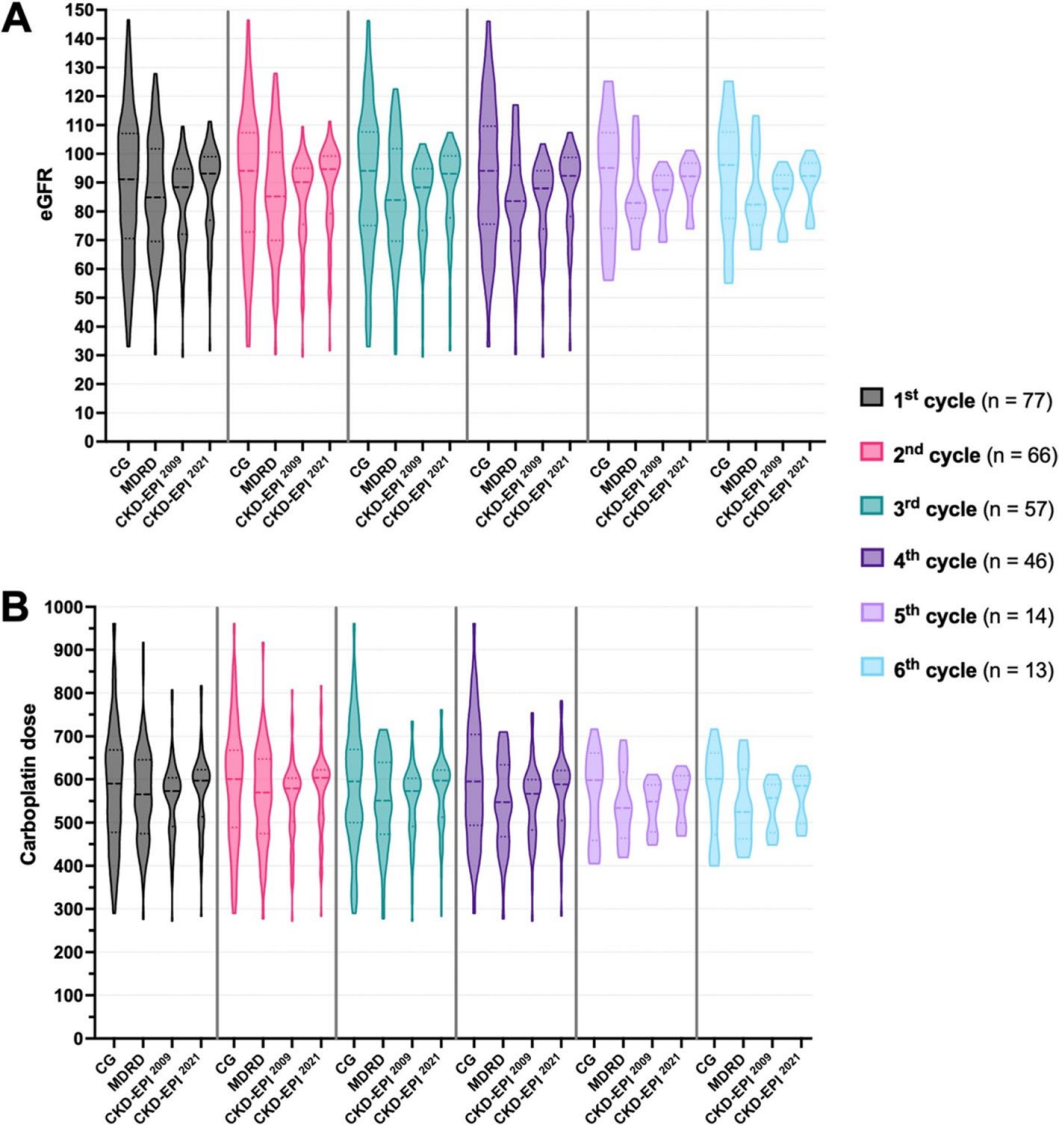
eGFR and carboplatin doses vary between different equations. **A **Violin graphs showing eGFR values in ml/min calculated by the CG, and ml/min/1.73 m^2^ calculated by the MDRD, CKD-EPI ^2009^, and CKD-EPI ^2021^. **B** Violin graphs showing carboplatin doses in mg for CG, and in mg/1.73 m^2^ for MDRD, CKD-EPI^2009^, and CKD-EPI^2021^ formulae. Values (eGFR or carboplatin dose) prior to each treatment cycle is indicated with a different colour. Median values are shown with dashed lines, and quartiles are shown with dotted lines. Only AUC-5/6 patients are included. See Supplementary Fig.1 for AUC-2 data

Once we determined the treatment protocols and patient characteristics, we calculated four different eGFR values for each patient across the six carboplatin cycles using the formulae listed in Fig. 1A. Since AUC-5/6 patients received significantly higher carboplatin doses than AUC-2 patients, we analysed these two groups separately (i.e., AUC-2 and AUC-5/6). Among the AUC-5/6 patients, the CG equation resulted in a broad range of eGFR values varying from approximately 30 to 150 ml/min for the first four carboplatin cycles (Fig. 3A). While slightly narrower, the MDRD equation also resulted in a wide eGFR range (30 to 130 ml/min/1.73 m^2^). On the contrary, both CKD-EPI ^2009^ and CKD-EPI ^2021^ yielded a markedly narrower eGFR range varying from 30 to 110 ml/min/1.73 m^2^. This is approximately a 33% reduction in the size of the eGFR scale. This range was particularly narrower for the 5^th^ and 6^th^ cycles (70 to 100 ml/min/1.73 m^2^) (Fig. 3A).

In accordance with patient eGFR values, at least 50% of patients would have received a carboplatin dose between 500 and 600 mg/1.73 m^2^ if their kidney function was estimated based on CKD-EPI ^2009^ or CKD-EPI ^2021^. However, the same patient group was prescribed a carboplatin dose between approximately 475 mg and 675 mg. The range of carboplatin doses was particularly narrower for the 5^th^ and 6^th^ cycles for almost all the patients. (Fig. 3B). Similar findings were observed for the AUC-2 patients where hypothetical carboplatin dosages calculated by CKD-EPI ^2009^ and CKD-EPI ^2021^ were within a smaller range than the actual CG-based eGFR and carboplatin values (Supp. Figure 1A and B).

### Significant differences between prescribed CG-based carboplatin dosages and hypothetical MDRD- and CKD-EPI-based carboplatin dosages

We aimed to determine how the CG-based carboplatin dosages would have changed if MDRD- or CKD-EPI-based eGFR values were applied in the Calvert formula. For this goal, we first calculated the percent dose change by substituting CG with any of the other three equations by the following simple formula:

$$\begin{array}{c}Dose\;change\;\left(\%\right)=\frac{\left(CG-based\;carboplatin\;dose\right)-\left(\varepsilon-based\;carboplatin\;dose\right)}{\left(CG-based\;carboplatin\;dose\right)}\times100\\\rangle\;\mathrm\varepsilon\;\mathrm i\mathrm s\;\mathrm a\mathrm n\mathrm y\;\mathrm o\mathrm f\;\mathrm M\mathrm D\mathrm R\mathrm D,{\mathrm{CKD}-\mathrm{EPI}}^{\;2009}\mathrm{or}\;{\mathrm{CKD}-\mathrm{EPI}}^{\;2021}\end{array}$$

**Fig. 4:**
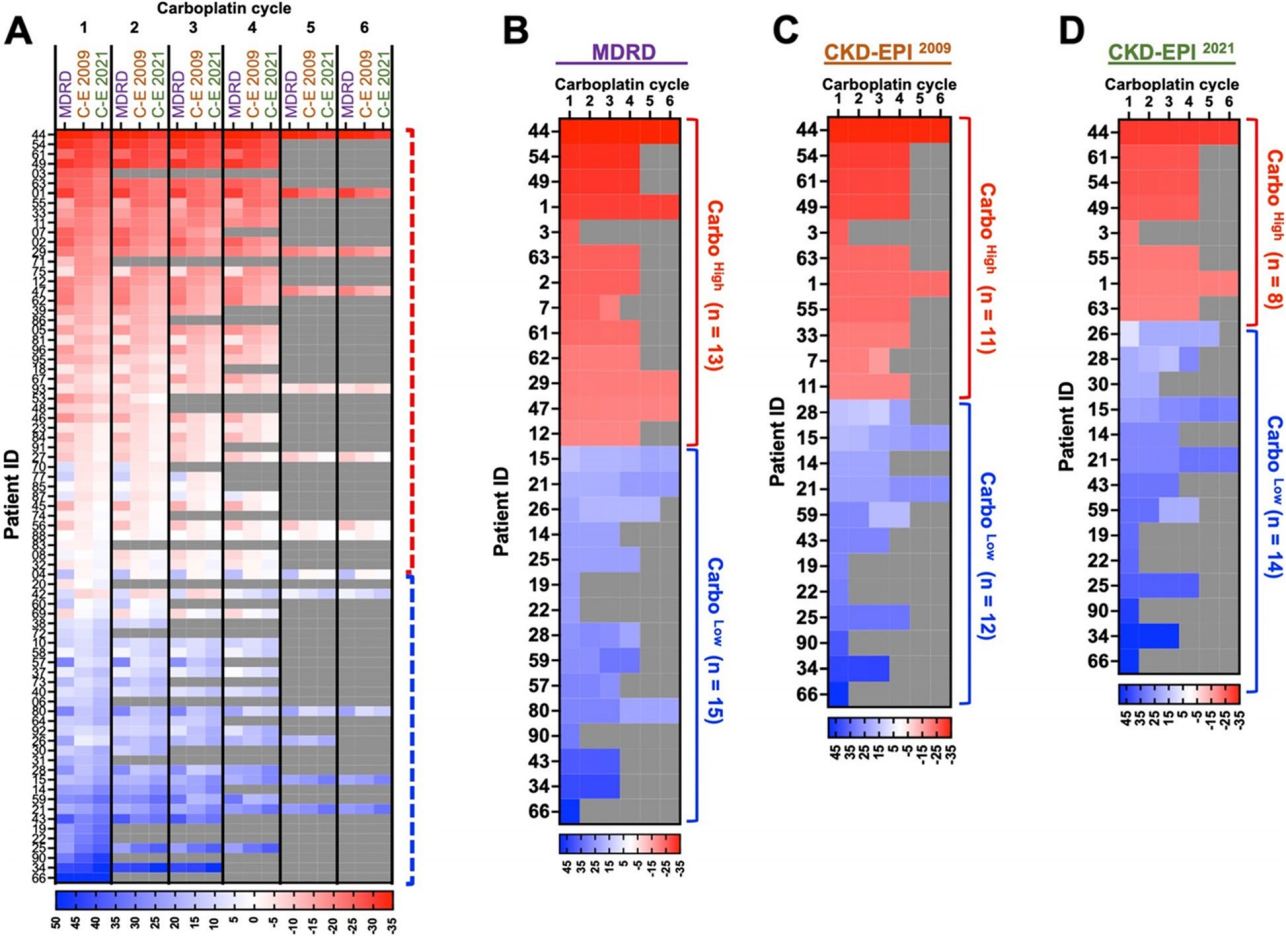
Marked differences between the prescribed carboplatin dosages and hypothetical carboplatin dosages.** A-D **MDRD-, CKD-EPI ^2009^-, and CKD-EPI ^2021^-based hypothetical carboplatin doses were compared to those that are based on the CG formula. Percent changes are displayed on heatmaps. Patients who would have received a lower dose based on MDRD, CKD-EPI ^2009^ and/or CKD-EPI ^2021^are shown with red boxes. Those who would have received a higher dose are shown with blue boxes. These two patient groups are indicated by vertical dashed or solid brackets. Grey boxes indicate a carboplatin cycle that was not administered. The intensity of the colours depicts the degree of change, and shown with a colour scale below the heatmaps. **A** Carboplatin changes for all AUC-5/6 patients. Carboplatin cycle numbers are displayed at the top of the heatmap. The name of each formula is indicated below the carboplatin cycle numbers. C-E 2009 and C-E 2021 stand for CKD-EPI ^2009^ and CKD-EPI ^2021^, respectively. **B-D** Carboplatin changes for the patients who would have received a dose that is at least 20% different than their original dose in any of the six carboplatin cycles as a result of substituting CG with MDRD (**B**), CKD-EPI ^2009^ (**C**), and CKD-EPI ^2021^ (**D**). Red boxes indicate Carbo ^High^ patients, and blue boxes indicate Carbo ^Low^ patients. See Supplementary Fig. 2 for AUC-2 data

This percent change was calculated for AUC-5/6 patients during all available carboplatin cycles and charted on a heatmap in which the colour intensity correlates with the degree of deviation from the CG-based carboplatin dose (Fig. 4A). Approximately two-thirds of the AUC-5/6 patients would have received a lower dose of carboplatin if CG was replaced by any of the MDRD, CKD-EPI ^2009^, or CKD-EPI ^2021^. The remaining one-third of the AUC-5/6 patients would have received a higher dose of carboplatin if CG was replaced by MDRD, CKD-EPI ^2009^ or CKD-EPI ^2021^ (Fig. 4A and Supp. Figure 2A-C).

We then focused on the patients who have shown the biggest degree of variation in either direction. For this goal, a threshold of 20% absolute change has been set, and the patients who would have experienced ≥ 20% change based on each eGFR formula were identified (Fig. 4B-D). Carbo^High^ patients were those who required at least 20% lower dose, and Carbo^Low^ patients were those who required at least 20% higher dose than that of CG-based calculations had estimated. More specifically, if CG was replaced by MDRD, 13 patients would have received at least 20% lower dose of carboplatin (Carbo^High^ patients), and 15 patients received at least 20% higher dose of carboplatin (Carbo^Low^ patients) in any of the six cycles (Fig. 4B and Supp Fig. 4A). A similar approach revealed that the number of Carbo^High^ patients was 11 and 8, and the number of Carbo^Low^ patients was 12 and 14 based on CKD-EPI ^2009^ and CKD-EPI ^2021^, respectively (Fig. 4C and D, and Supp. Figure 2B and C). Notably, more than half of these patients were common among all three formulae, reaching up to 80% overlap between CKD-EPI ^2009^ and CKD-EPI ^2021^. These findings suggest that approximately 10–17% of the AUC-5/6 patients had received a carboplatin dose that was at least 20% higher than a non-CG formula would have predicted, and 16–20% of the AUC-5/6 patients received a dose that was at least 20% lower than a non-CG formula would have predicted.

When a similar strategy was applied to AUC-2 patients, the range of carboplatin dose change was less pronounced when compared to those of AUC-5/6 patients. Of the 19 AUC-2 patients, only 2–4 of them would have received a carboplatin dose that was at least 20% different than the CG-based dose if any of MDRD, CKD-EPI ^2009^ or CKD-EPI ^2021^ was utilised (Supp. Figure 2D). Given the fact that AUC-2 patients receive a significantly lower dose of carboplatin, utilisation of any of the eGFR formulae would result in comparable dosages.

### Bodyweight and body surface area have a significant effect on CG-based eGFR and carboplatin values

**Fig. 5:**
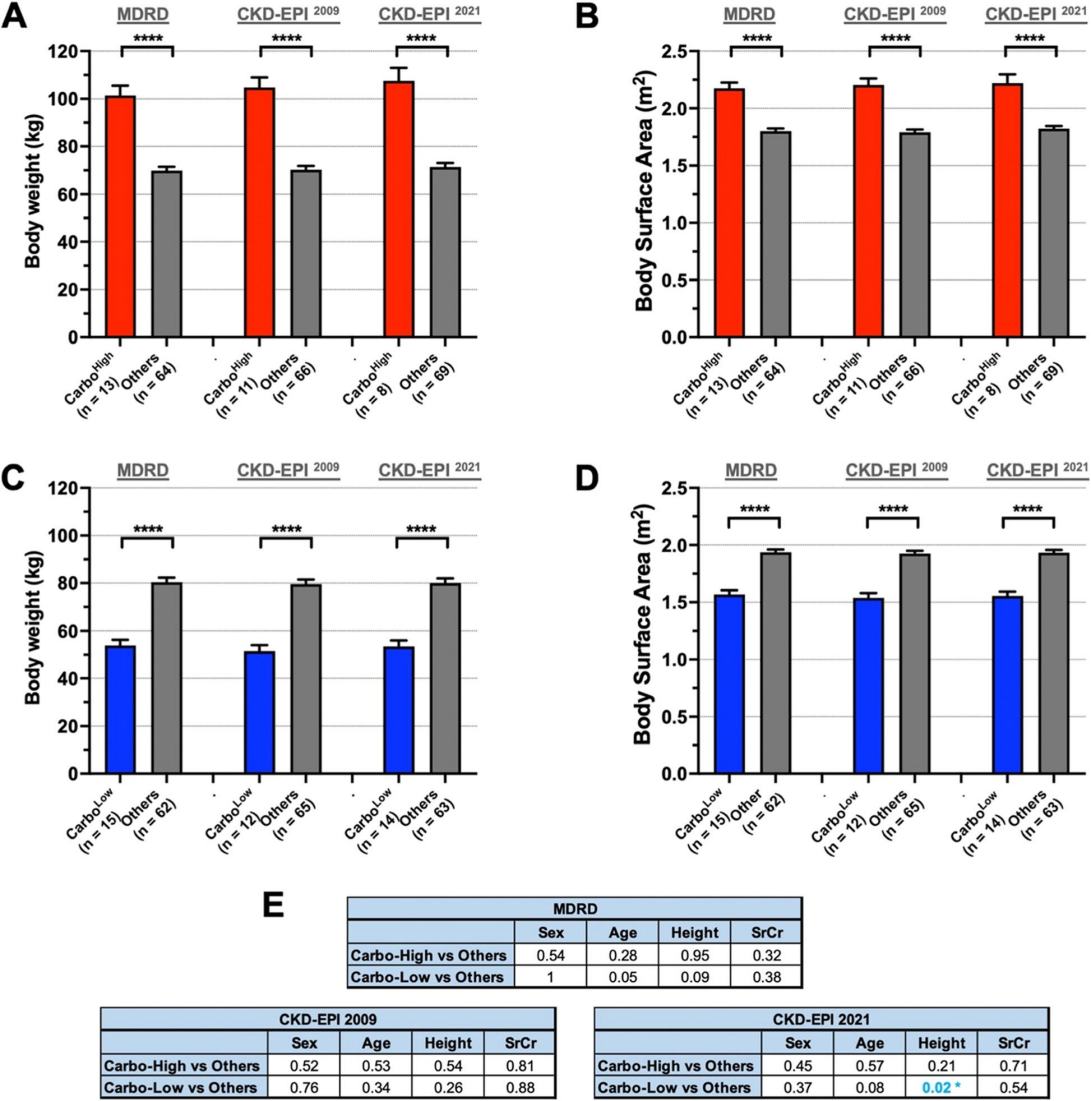
Comparison of baseline patient characteristics between different patient groups.** A-D** Patient characteristics (i.e., body weight and body surface area (BSA)) are compared between Carbo ^High^ patients (red bars) and Others (grey bars) (**A **and** B**), or Carbo ^Low^ patients (blue bars) and Others (grey bars) (**C** and **D**) who were identified previously in Fig. 4. Body weight comparisons are shown in (**A**) and (**C**); BSA comparisons are shown in (**B**) and (**D**). The name of the formulae that have identified these patient groups are indicated at the top of the bars. **E** Sex, age, height, and SrCr values are compared between Carbo^High^ patients and Others or Carbo^Low^ patients. The name of the formulae that have identified these patient groups are indicated at the top each table. Fisher’s exact test is used for sex comparison; unpaired, two-tailed t-test is used for all other statistical comparisons. * indicates a *p*-value < 0.05, **** indicates a *p*-value < 0.0001. See Supplementary Fig. 3 for comparisons among AUC-2 patients

After identifying the AUC-5/6 patients whose carboplatin doses were most significantly affected by employing an eGFR formula different from CG, we investigated the patient characteristics most responsible for this change. We first compared the bodyweight and body surface area (BSA) between patients who had at least 20% change (i.e., Carbo^High^ and Carbo^Low^) with those without such change as a result of replacing the CG formula. When Carbo^High^ patients were compared to the remainder of the patient cohort (i.e., “Others”), we found that Carbo^High^ patients had significantly higher bodyweight and BSA regardless of the formula that replaced CG (Fig. 5A and B, respectively). Conversely, Carbo^Low^ patients had significantly lower bodyweight and BSA compared to the remainder of the cohort (Fig. 5C and D, respectively). Other patient characteristics, including sex, age, and SrCr value did not have any significant impact on carboplatin dose change by replacing CG with any of the MDRD, CKD-EPI ^2009^, and CKD-EPI ^2021^ (Fig. 5E). Height seemed to have an impact only when CG was replaced with CKD-EPI ^2021^ equation; however, this is arguably associated with bodyweight (Fig. 5E).

We then applied the same comparisons for AUC-2 patients. Since there was a maximum of four AUC-2 patients who would have experienced at least 20% change in consequence of replacing the CG formula (Supp. Figure 2D), we lowered the threshold to 10% to be able to have a statistically sufficient number of patients. Thus, Carbo^High^ patients were those who would have received at least a 10% reduction, and conversely, Carbo^Low^ patients were those who would have received at least a 10% increase in their carboplatin dose if CG was replaced by the other equations. AUC-2 findings were similar to those of AUC-5/6 in that both bodyweight and BSA had a significant impact on carboplatin dose change by replacing the CG formula (Supp. Figure 3A-D). Interestingly, Carbo^Low^ patients, who theoretically needed a higher carboplatin dose, had significantly lesser height in AUC-2 patients when CG was replaced with any of MDRD, CKD-EPI 2009 or CKD-EPI ^2021^ equations (Supp. Figure 3E). This finding is consistent with the impact of bodyweight in CG-based carboplatin calculation. Lastly, sex, age, and SrCr value did not have any significant impact on carboplatin dose change in AUC-2 patients by replacing CG with other formulae (Supp. Figure 3F).

These findings suggest that the CG equation overestimates kidney function, and thus carboplatin clearance, in patients with a higher bodyweight and BSA while underestimating such parameters in patients with a lower bodyweight and BSA.

### CKD-EPI formulae estimations correlate with clinical assessment and carboplatin dose adjustment

**Fig. 6:**
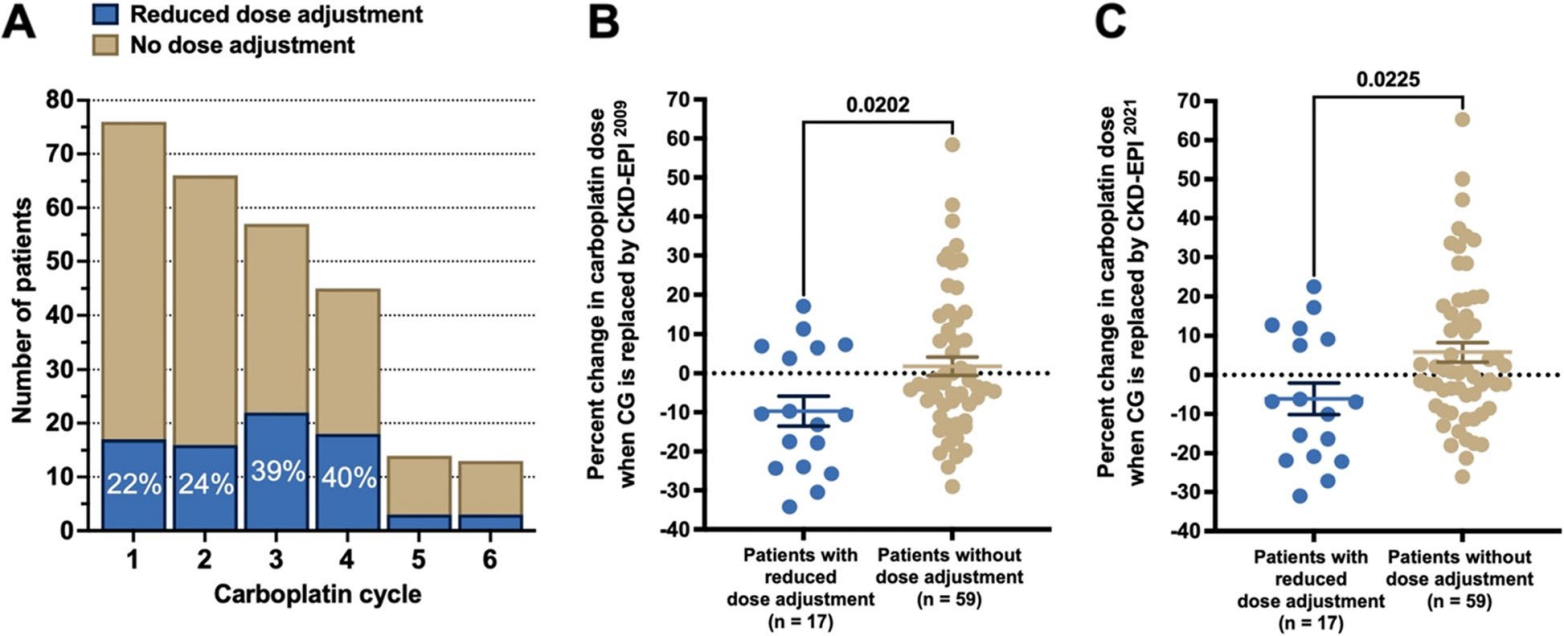
Hypothetical carboplatin doses were re-evaluated within the clinical context.The patients who received a clinically reduced carboplatin dose would have received a lower dose upfront if CKD-EPI ^2009^ or CKD-EPI ^2021^ was used instead of CG in eGFR calculation. **A **The number of patients whose carboplatin dose was reduced from the original calculated dose based on their clinical presentation (e.g., ECOG performance status, observations, and subjective medical officer assessment). **B **and** C** The patients who received a clinically-reduced carboplatin dose were compared to those without dose adjustment in terms of their hypothetical carboplatin dose change as a result of substituting CG with CKD-EPI ^2009^ (**B**) or CKD-EPI ^2021^(**C**). Data acquired from the 1^st^ cycle of carboplatin treatment

To determine whether carboplatin dose reductions made due to patient clinical deterioration correlated with suboptimal initial dosing, we first identified the patients whose carboplatin dose was reduced by the medical officer prior to its administration on the treatment day. We observed a progressive increase in the fraction of patients whose carboplatin dose was reduced throughout the first four cycles of the treatment (Fig. 6A).

We then investigated whether any of the eGFR equations would have predicted a lower dose for these patients before they were given the treatment. For this purpose, we performed the analysis prior to the first carboplatin cycle, during which the patients were not exposed to any carboplatin treatment. Interestingly, both CKD-EPI ^2009^ and CKD-EPI ^2021^ would have estimated a significantly lower carboplatin dose for these patients with reduced dose adjustment compared to those whose carboplatin dose was not changed on the treatment day (Fig. 6B and C). We did not detect a similar clinical correlation for the MDRD equation, emphasizing the advantage of using CKD-EPI equations over MDRD (Supp. Figure 4). These findings suggest that the patients who received a clinically reduced carboplatin dose would have received a lower dose upfront if CKD-EPI ^2009^ or CKD-EPI ^2021^ was used instead of CG in eGFR calculation.

### CKD-EPI^2021^ is a simplified version of the CKD-EPI^2009^ formula

**Fig. 7:**
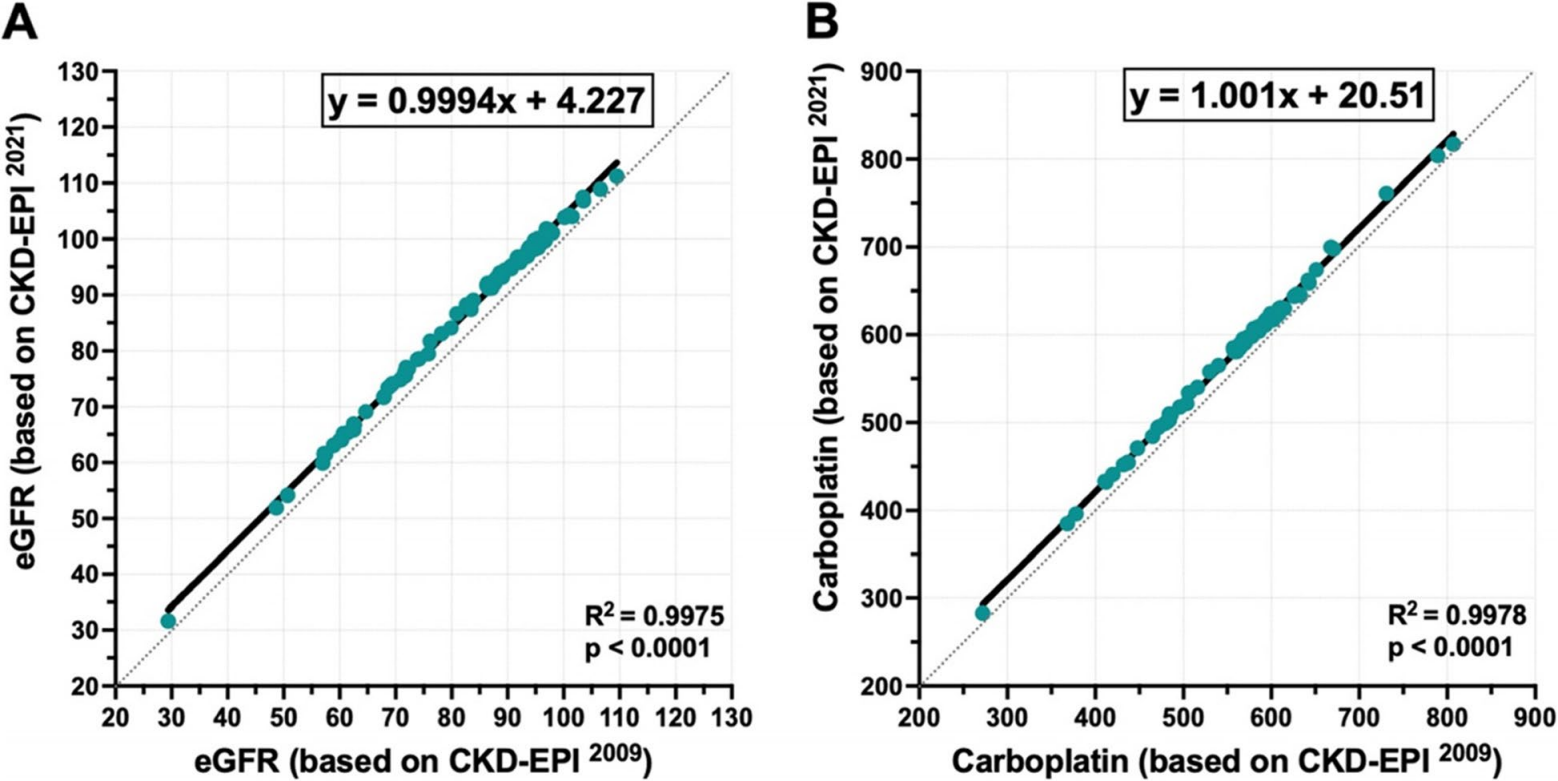
Simple linear regression models are used to describe the relationship between CKD-EPI^2009^(x-axes) and CKD-EPI ^2021^(y-axes) equations. The relationship based on eGFR values is shown in (**A**) and the relationship based on corresponding carboplatin dosages is shown in (**B**). The data from all AUC-2,-5,-6 patients prior to the first carboplatin cycle (*n* = 96) were used in (**A**) and the data from AUC-5/6 patients prior to the first carboplatin cycle (*n* =77) were used in (**B**). The relationships are described by linear formulae, the *p*-values of the relationships and R^2^ values are indicated

Since CKD-EPI ^2009^ and CKD-EPI ^2021^ formulae resulted in very comparable findings in the current study, we aimed to define the relationship between these two versions in mathematical equations. The eGFR values (Fig. 7A) and the corresponding carboplatin dosages (Fig. 7B) prior to the first carboplatin cycle were plotted in separate graphs. Simple linear regression models suggested a statistically significant correlation between the two versions of CKD-EPI formulae, which is defined as;

$$\begin{array}{c}y=0.994x+4.227\left(for\;eGFR\right)\\y=1.001x+20.51\left(for\ carboplatin\ dose\right)\\\rangle\;x\;\mathrm{is}\;{\mathrm{CKD}-\mathrm{EPI}}^{2009}\mathrm{and}\;y\;\mathrm{is}\;{\mathrm{CKD}-\mathrm{EPI}}^{2021}\end{array}$$

Based on these mathematical relationships, the CKD-EPI ^2021^ version estimates approximately 4.2 ml/min/1.73 m^2^ higher eGFR than the original CKD-EPI ^2009^ version (Fig. 7A). Consequently, the carboplatin dose would differ approximately 8 mg (for an AUC-2 patient) to 21 mg (for AUC-5/6 patients) between two equations (Fig. 7B and Supp. Figure 5A). These findings suggest that CKD-EPI ^2021^ formula functions as accurately as CKD-EPI ^2009^ in calculation of eGFR and carboplatin. Notably, Black patients with high eGFR values can experience a bigger change in their eGFR and carboplatin dose as a result of omitting the race coefficient in CKD-EPI ^2021^ formula (e.g., up to 15 ml/min/1.73 m^2^ and 90 mg reduction, respectively), which may require specific consideration to these patient groups. (Supp. Figure 5B and C) [30].

### BSA adjustment of eGFR affects carboplatin dosing in patients with specific baseline characteristics

**Fig. 8:**
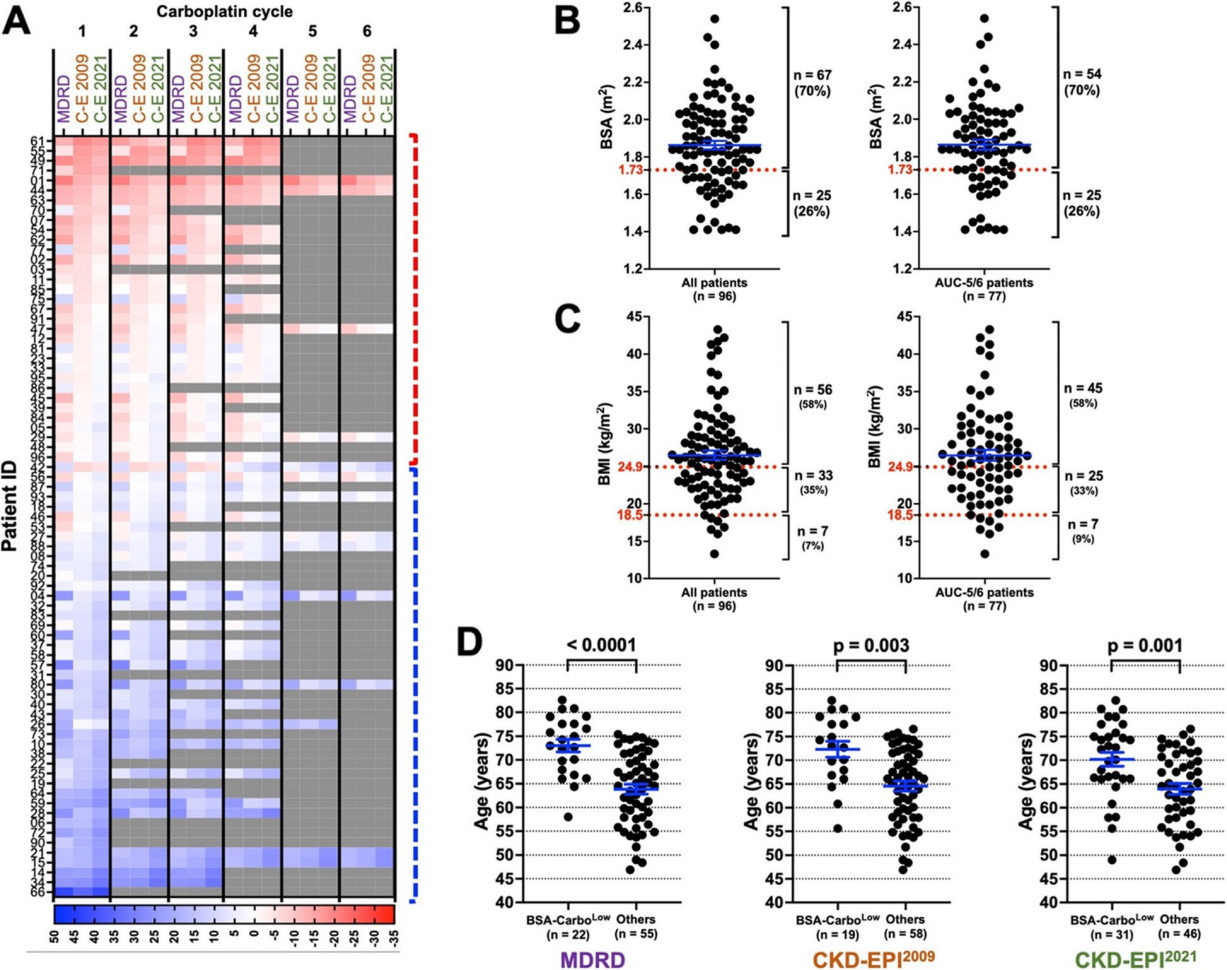
BSA adjustment reduces the differences between the CG and the MDRD-, CKD-EPI ^2009^-, or CKD-EPI ^2021^ equations. **A** BSA-adjusted MDRD-, CKD-EPI ^2009^-, and CKD-EPI ^2021^-based hypothetical carboplatin doses were compared to those that are based on the CG formula. Carboplatin percent changes are shown on heatmaps. Patients who would have received a lower dose based on MDRD, CKD-EPI ^2009^ and/or CKD-EPI ^2021^are shown with red boxes. Those who would have received a higher dose are shown with blue boxes. These two patient groups are indicated by vertical dashed or solid brackets. Grey boxes indicate a carboplatin cycle that was not administered. The intensity of the colours depicts the degree of change, and shown with a colour scale below the heatmaps. Carboplatin cycle numbers are displayed at the top of the heatmap. The name of each formula is indicated below the carboplatin cycle numbers. C-E 2009 and C-E 2021 stand for CKD-EPI ^2009^ and CKD-EPI ^2021^, respectively. **B** Scatter plots showing the BSA of all patients (left) and of AUC-5/6 patients only (right). **C** Scatter plots showing the BMI of all patients (left) and of AUC-5/6 patients only (right). **D** Age distribution of BSA-Carbo^Low^ patients and the rest of the cohort. Older age is a key determinant in BSA-adjusted carboplatin dosing. Mean and SEM is shown with blue lines in all graphs. 1.73 m2 BSA and healthy BMI (i.e., 18.5-24.9) are shown with dotted red lines

Previous studies suggested BSA adjustment for the MDRD and CKD-EPI-generated eGFR in order to account for the unit differences between GFR estimation formulae (i.e., ml/min vs ml/min/1.73 m^2^ for eGFR and mg vs mg/1.73 m^2^ for carboplatin dose) [21, 31]. This was achieved by multiplying MDRD or CKD-EPI-derived eGFR by BSA/1.73 for each patient. Accordingly, the differences between the CG and MDRD or CKD-EPI equations were less pronounced in BSA-adjusted eGFR and carboplatin doses (Supp. Figure 6A and B). Furthermore, the Carbo^High^ group virtually disappeared as a result of BSA adjustment, and the number of Carbo^Low^ patients reduced markedly, particularly in the CKD-EPI ^2009^ substitution of the CG (Supp. Figure 6C-E). Therefore, we made an ad hoc reduction in the threshold of the carboplatin percent change from 20 to 10% to define the new Carbo^Low^ and Carbo^High^ patients (BSA-Carbo^Low^ and BSA-Carbo^High^ from hereon). This strategy allowed us to better identify and compare the baseline characteristics of AUC-5/6 patients who were most affected by the BSA-adjusted carboplatin dosing. Interestingly, the number of BSA-Carbo^Low^ patients was strikingly higher than the number of BSA-Carbo^High^ patients as a result of BSA adjustment, particularly for the CKD-EPI^2021^ equation where the BSA-Carbo^High^:BSA-Carbo^Low^ ratio was 1:6.2 (Fig. 8A, Supp. Figure 7A-C). This contrasts with our previous finding without the BSA-adjustment where Carbo^High^ and Carbo^Low^ group sizes were more comparable (e.g., 1:1.75 in CKD-EPI ^2021^) (Fig. 4B, C, and D). This is a likely consequence of the high BSA and high BMI profile of our patient population resulting in a higher eGFR estimate for the patients. 70% of the patients had a BSA > 1.73 m^2^ (mean = 1.86 m^2^) and 58% of the patients had a BMI > 24.9 kg/m^2^ (mean = 26.47 kg/m^2^) (Fig. 8B and C).

We then aimed to determine the patient characteristics that can lead to carboplatin overtreatment or undertreatment in the new BSA-adjusted carboplatin dosing. When we analysed the patient characteristics between the BSA-Carbo^High^ and BSA-Carbo^Low^ group, we found similar results to the non-BSA-adjusted data. BSA-Carbo^High^ patients had significantly higher bodyweight and BSA, and BSA-Carbo^Low^ patients had significantly lower bodyweight and BSA compared to the remainder of the cohort (Supp. Figure 8A-D). BSA-Carbo^Low^ patients were consistently found to have significantly older age than the rest of the patient cohort, suggesting that CG-derived carboplatin dosing might lead to reduced therapeutic effect in patients with older age (Fig. 8D). It is worth noting that older age was not a key determinant of the eGFR formulae that lead to significant differences in non-BSA-adjusted carboplatin dosing (Fig. 5E). Sex (female), and lower height were significantly associated with the BSA-Carbo^High^ patient group; however, these results should be interpreted with caution due to small sample size of the BSA-Carbo^High^ group (Supp. Figure 8E). Taken together, these data suggest that clinical assessment of specific patient characteristics remains fundamental even when BSA adjustment is applied to the MDRD- or CKD-EPI-derived eGFR values prior to their use in the Calvert formula.

## Discussion

Carboplatin, like many cytotoxic agents, is almost exclusively eliminated renally and has a narrow therapeutic index, causing potentially major adverse reactions with subtle dose changes. Previous studies suggested that a carboplatin dose reduction as small as 10% may result in a doubling of the 5-year relapse rate [21]. Therefore, accurate dosing based on renal clearance and drug exposure is critical to ensure patient safety while maintaining the highest achievable therapeutic goal. The CG formula is still commonly used to estimate renal function in patients with cancer globally, despite its relative shortcomings compared to more contemporary Eqs. [21]. This is partly because the recommendations from the 2012 Kidney Disease Improving Global Outcomes (KDIGO) and the National Kidney Foundation-Kidney Disease Outcomes Quality Initiative (NKF-KDOQI) guidelines have not been fully adopted by several non-nephrology specialities, including oncology [21]. Other reasons might include; convenience in the use of CG due to its well-established implementation within the healthcare systems, CG’s perceived accuracy, lack of convincing evidence to adopt a new guideline or standard, and insufficient illustration of the potential clinical impacts of substituting CG with more modern equations. Therefore, our study investigated the impact of different eGFR formulae on carboplatin dose calculations, and whether these differences had clinical implications in the treatment of patients with lung cancer within the confines of a retrospective analysis.

We compared four different equations (i.e., CG, MDRD, CKD-EPI ^2009^ and CKD-EPI ^2021^) using data from patients with SCLC, NSCLC, and mesothelioma. Our findings indicated that CKD-EPI-based equations resulted in a narrower spectrum of eGFR values. CG-based carboplatin dosages differed markedly between patients at the extremities, receiving the highest or lowest carboplatin doses. Our results showed that up to 14% of patients were treated with at least 20% higher carboplatin dose than a CKD-EPI equation would have predicted. Conversely, 18% of the patients were treated with at least 20% lower carboplatin dose than a CKD-EPI equation would have predicted. Taken together, this suggests that almost one-third of the whole patient cohort received a sub-optimal carboplatin dose.

Patient stratification according to baseline characteristics can be an essential clinical tool to identify patients who are most affected by employing different eGFR formulae in carboplatin dose calculation. Our findings suggested that CG-based eGFR overestimates kidney function in patients with higher bodyweight and BSA, whilst underestimating it in patients with lower bodyweight and BSA. Notably, CG was generated in the early 70s, and it is the only equation tested in this study that uses bodyweight as a parameter. However, there have been substantial changes in the BMI profile of the world population from the early 70s to the current date. Recent comprehensive studies found that age-standardised mean BMI increased from 22.1 kg/m^2^ in 1975 to 24.8 kg/m^2^ kg/m^2^ in 2016 in women, and from 21.7 kg/m^2^ in 1975 to 24.5 kg/m^2^ kg/m^2^ in 2016 in men confirming the well documented increased prevalence of being overweight between these two periods [32, 33]. This further supports that the CG formula is outdated and no longer fit for purpose, and CG should be adjusted to account for the extremities of patient characteristics, including obesity and age.

The 2012 KDIGO clinical practice guidelines and the NKF-KDOQI guideline groups now recommend CKD-EPI equations in eGFR calculation [20, 27, 28]. In addition to its impact on carboplatin dosing, we also assessed whether there was clinical evidence that supports the use of CKD-EPI equations instead of CG or MDRD. Our results showed that CKD-EPI equations would have estimated lower carboplatin doses for patients whose pre-determined CG-based carboplatin doses were reduced by the attending medical officer based on patients’ clinical presentation. This observation suggests that, as expected, CKD-EPI provides a more reliable eGFR estimation that is safer in reducing carboplatin-related toxicity. Interestingly, the association between CKD-EPI estimations and clinical decisions was detected only for the first carboplatin cycle, but not the remainder of the treatment schedule. This perhaps could be explained by insufficient patient number to reliably power this assessment or be due to reduced eGFR values that already translated into lower carboplatin dosages for successive cycles.

The CKD-EPI ^2009^ creatinine equation was recently updated to the CKD-EPI ^2021^ version, with the major change being the omittance of a race coefficient from the former version. Ideally, public health and clinical guidelines should be inclusive of all races, and medical practice should not rely on complex traits, such as race, as a major determinant. Previous studies observed a higher GFR value in Black patients than non-Black patients of a similar age, the same sex, and similar SrCr value. This difference was reflected in CKD-EPI ^2009^ equation with a race coefficient that estimates approximately 15.9% higher eGFR values for Black patients. Therefore, removing the race in CKD-EPI ^2021^ equation resulted in a lower eGFR value for Black patients causing the exclusion of more Black patients from receiving anticancer therapy [30]. With these principles and findings in mind, we compared the two versions of CKD-EPI and found a strong relationship between them. Based on our data, using either of the two CKD-EPI versions would make approximately 8–21 mg/1.73 m^2^ difference in carboplatin dose calculation for non-Black patients. This difference would, however, be much higher (e.g., > 90 mg/1.73 m^2^ reduction in carboplatin) for Black patients, raising questions whether they would be receiving a subtherapeutic carboplatin dosage. Therefore, a deeper understanding of how the estimation of kidney function affects the patient outcome is still necessary while supporting the utilisation of a simpler and more inclusive equation without a compromise in clinical standards.

The CG formula uses bodyweight as a direct parameter, and it is not indexed for BSA. Therefore, this formula is not accurate for patients with extremes of bodyweight as it falsely overestimates kidney function in overweight/obese patients while underestimating it in underweight patients, particularly thin elderly. The newer formulae, including MDRD, CKD-EPI ^2009^ and CKD-EPI ^2021^, are indexed for the BSA, and their eGFR is expressed in ml/min/1.73 m^2^, accounting for this discrepancy caused by the wide spectrum of bodyweights in the current patient population. Therefore, CKD-EPI equations, particularly, are emerging as the most appropriate to use in daily clinical practice. BSA-indexing can then be removed by multiplying MDRD and CKD-EPI-derived eGFR values by BSA/1.73 m^2^ prior to their use in Calvert formula to calculate the carboplatin dose. Our findings indicated that BSA adjustment reduced the differences in eGFR and thus carboplatin dose between the CG and MDRD or CKD-EPI equations. Since BSA is directly correlated with the bodyweight, these findings highlight the issue of whether BSA adjustment re-introduced the impact of bodyweight that was observed in CG. This is particularly important for our modern patient population with increasing bodyweight and BSA, as 70% of our patient cohort had a BSA > 1.73 m^2^ and 58% of the cohort had a BMI > 24.9 kg/m^2^. Furthermore, BSA-adjusted data suggested that the eGFR of the older patients is underestimated based on the CG calculation. These patients received at least 10% less carboplatin dose than the BSA-adjusted MDRD and/or CKD-EPI equations would have predicted. Therefore, the impact of the BSA adjustment of carboplatin dosing on elderly patients should be determined accurately in order to avoid subtherapeutic treatment protocols. On the other hand, female and/or shorter patients received at least 10% higher dose than the MDRD and/or CKD-EPI equations would have predicted. Of course, these findings suggest that even the best eGFR formula may not be sufficient to account for all differences in patient physique and physiological function. Therefore, it is imperative that the current GFR estimation formulae should be used with caution while close monitoring of patient outcomes should lead the clinical decision in adopting the most suitable eGFR algorithm. Furthermore, these findings highlight the need for improved eGFR formulae that incorporate specific patient outcomes and biological/medical background.

In conclusion, here we compared different eGFR equations and illustrated their individual impacts on carboplatin dosing in the treatment of patients with lung cancer. We identified the baseline patient characteristics that are associated with drug toxicity and optimal treatment efficacy. We then investigated the relationship between our theoretical and clinical findings, and lastly, investigated the impact of CKD-EPI updates on chemotherapy dosing. To the best of our knowledge, the current is study is unique in analysing four different eGFR equations and their respective carboplatin dosages across multiple treatment cycles. Lastly, it is important to note that the findings in the presented retrospective study are based on the comparison of estimated GFR derived from different mathematical formulae without controls and actual measurements of GFR or blood concentration levels of carboplatin. The results should be interpreted with the consideration of these limitations.

## Supplementary Information

**Additional file 1.**

**Additional file 2.**

## Data Availability

The dataset(s) supporting the conclusions of this article is(are) included within the article (and its additional file(s)).
