# Supplementary material for: Carboplatin dose calculations for patients with lung cancer: significant dose differences found depending on dosing equation choice
Source: BMC Cancer. 2022 Jul 30;22:829. doi: 10.1186/s12885-022-09885-7 (PMC9338596; doi:10.1186/s12885-022-09885-7)
Supplement: Supplementary file 1 — Additional file 1. [file 12885_2022_9885_MOESM1_ESM.docx]

*Supplementary Material*

**Carboplatin Dose Calculations for Patients with Lung Cancer: Significant Dose Differences Found Depending on Dosing Equation Choice**

Seçkin Akgül, Bryan A Chan, and Peter M Manders


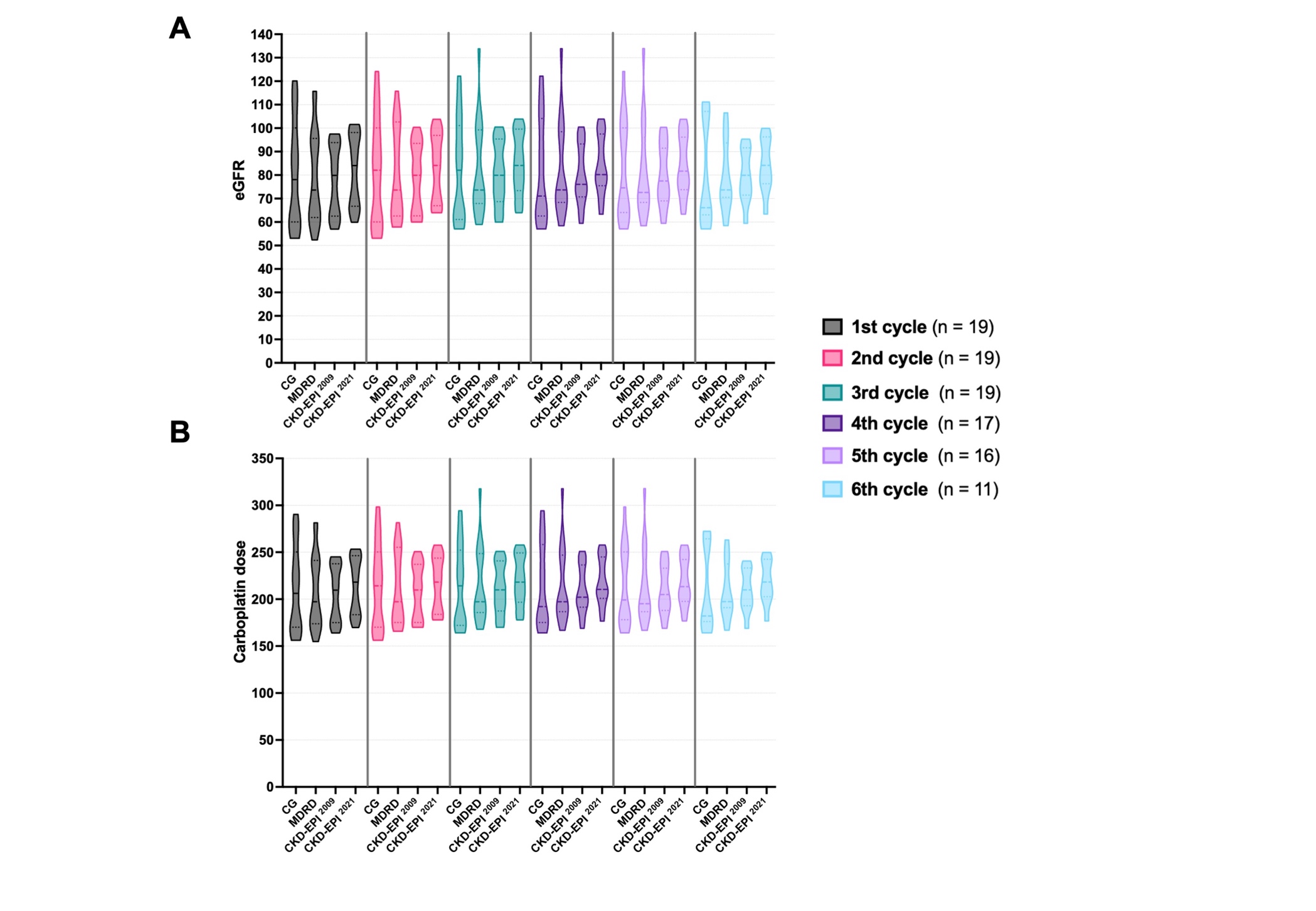


***Supplementary Fig. 1: A*** *Violin graphs showing eGFR values in ml/min calculated by the CG, and ml/min/1.73 m^2^ calculated by the MDRD, CKD-EPI^2009^, and CKD-EPI^2021^ formulae.* ***B*** *Violin graphs showing carboplatin doses in mg for CG, and in mg/1.73 m^2^ for MDRD, CKD-EPI^2009^, and CKD-EPI^2021^. Values (eGFR or carboplatin dose) prior to each treatment cycle is indicated with a different colour. Median values are shown with dashed lines, quartiles are shown with dotted lines. Only AUC-2 patients are included.*


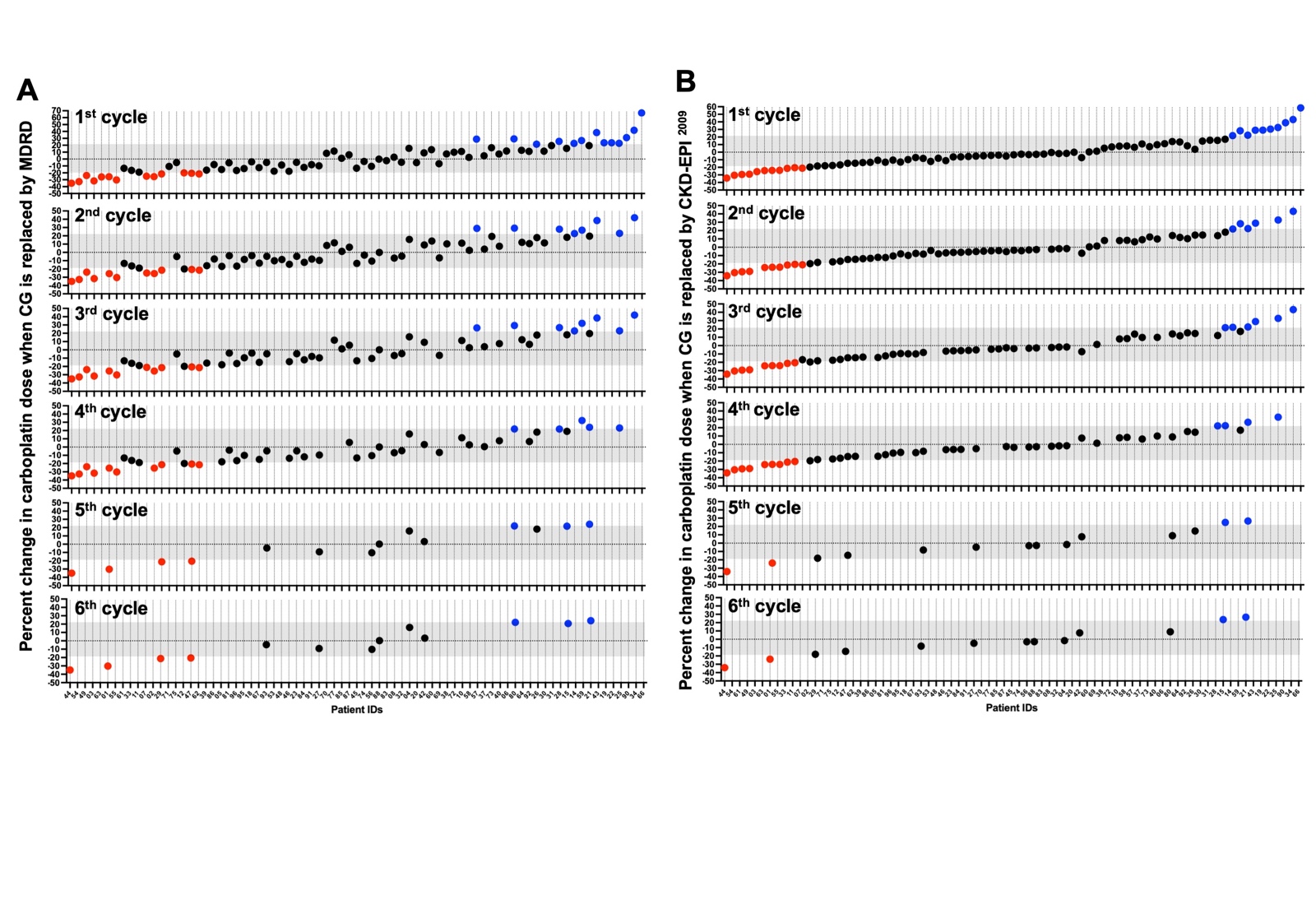


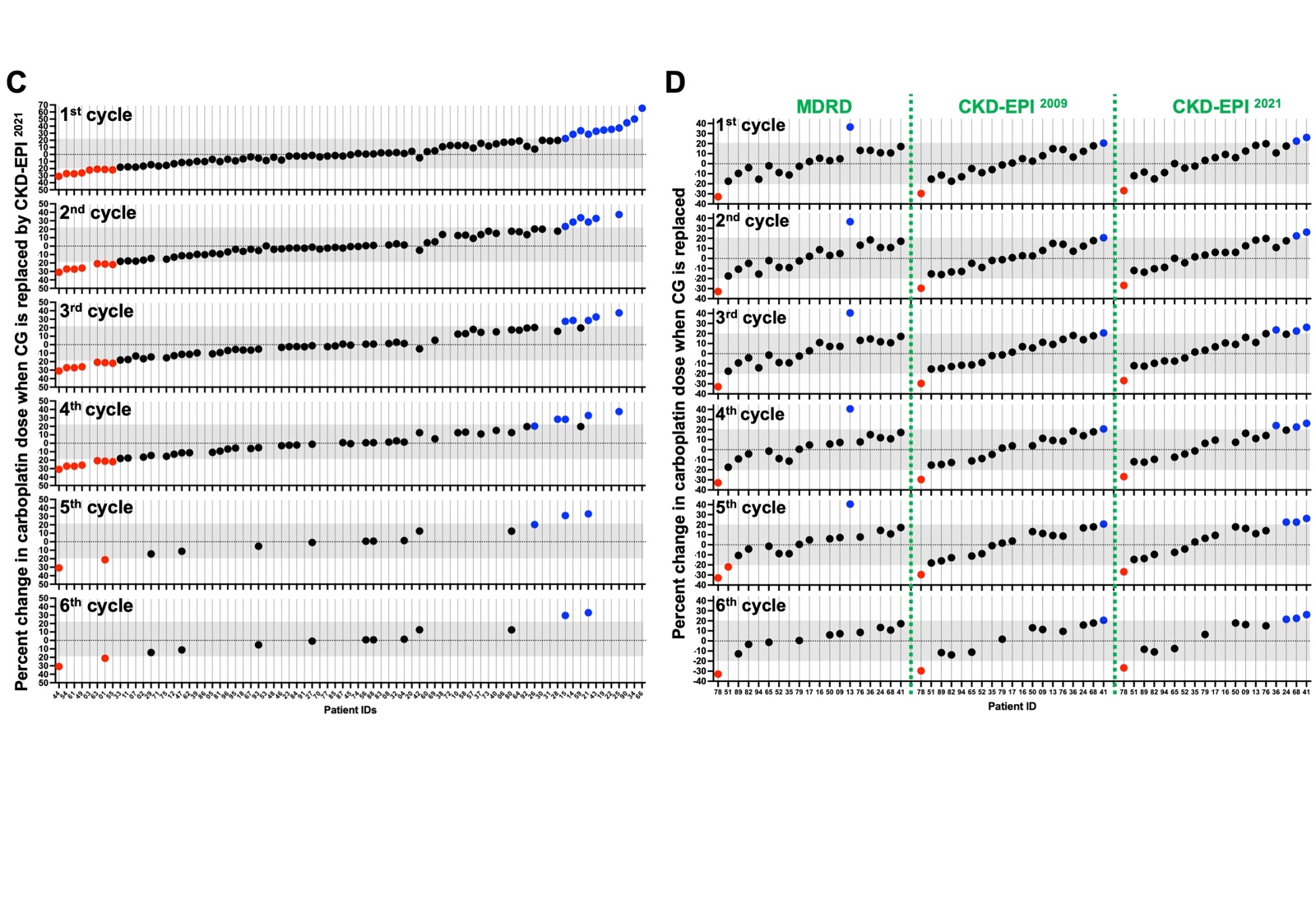


***Supplementary Fig. 2: A-C*** *MDRD-, CKD-EPI ^2009^-, and CKD-EPI ^2021^-based hypothetical carboplatin doses were compared to those that are based on CG formula. Percent changes are shown for MDRD* ***(A),*** *CKD-EPI ^2009^* ***(B)****, and CKD-EPI ^2021^****(C)****. Patients who would have received at least 20% lower dose based on MDRD, CKD-EPI ^2009^ and/or CKD-EPI ^2021^ are shown with red dots (Carbo^High^ patients). Those who would have received at least 20% higher dose are shown with blue dots (Carbo^Low^ patients). Remaining patients (neither Carbo^High^ nor Carbo^Low^) are shown with black dots in a grey region. Carboplatin cycle numbers are indicated on the left side of each graph. Data from AUC-5/6 patients was used.* ***D*** *Carboplatin percent changes as a result of substituting CG with MDRD, CKD-EPI ^2009^ or CKD-EPI ^2021^ are shown for AUC-2 patients. Changes based on each eGFR formula is depicted side-by-side and separated by a green dashed line.*


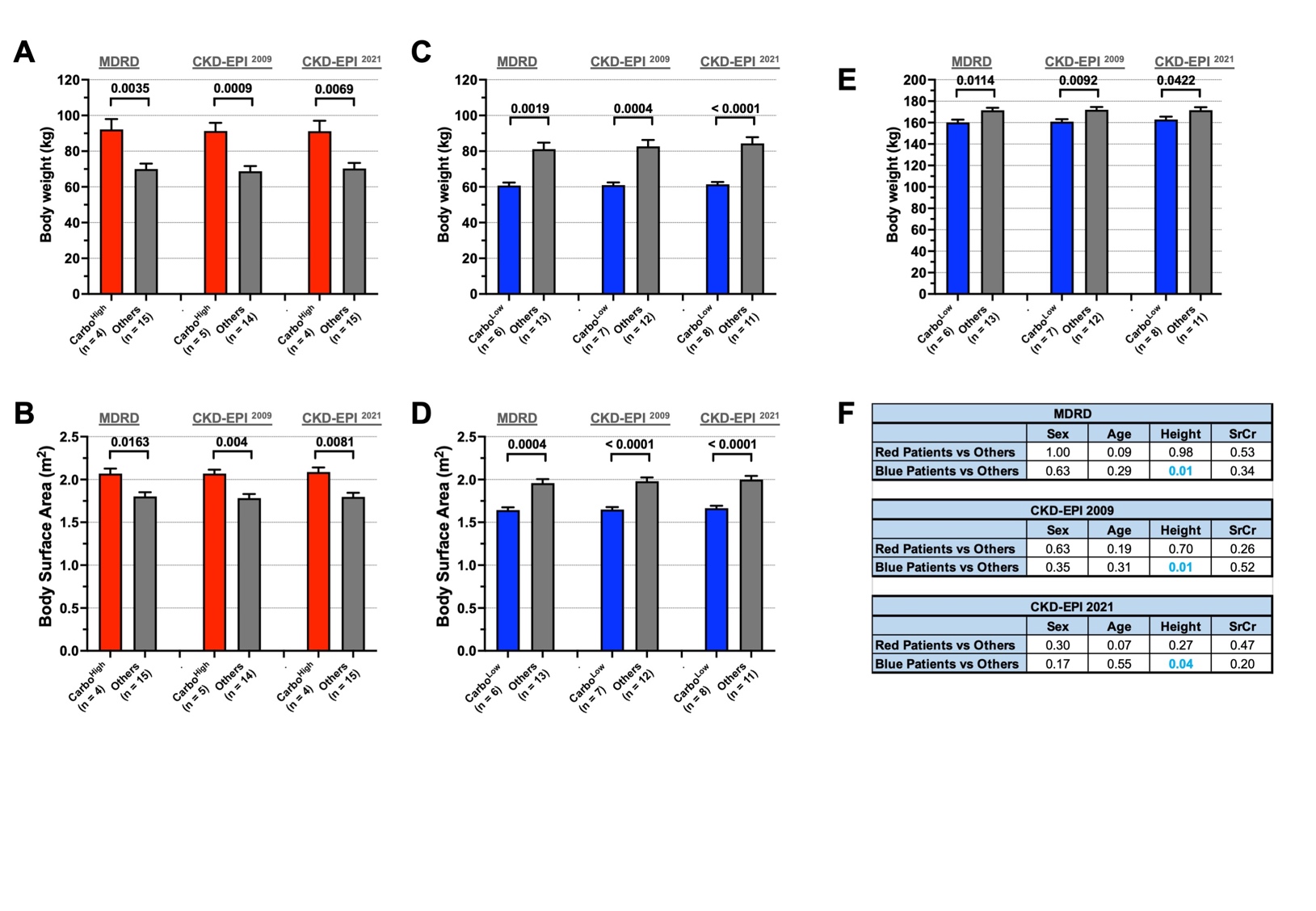


***Supplementary Fig. 3: A-D*** *Patient characteristics (i.e., body weight and body surface area (BSA)) are compared between Carbo ^High^ patients (red bars) and Others (grey bars)* ***(A and B)****, or Carbo ^Low^ patients (blue bars) and Others (grey bars)* ***(C and D)*** *who were identified previously in Fig. 4. Body weight comparisons are shown in* ***(A)*** *and* ***(C)****; BSA comparisons are shown in* ***(B)*** *and* ***(D)****.* ***E*** *Height is compared between Carbo ^Low^ patients (blue bars) and Others (grey bars). The name of the formulae that have identified these patient groups are indicated at the top of the bars.* ***F*** *Sex, age, height, and SrCr values are compared between Carbo^High^ patients and Others or Carbo^Low^ patients. The name of the formulae that have identified these patient groups are indicated at the top each table. Fisher’s exact test is used for sex comparison; unpaired, two-tailed t-test is used for all other statistical comparisons. **** indicates a p-value < 0.0001. Data from AUC-2 patients were used.*


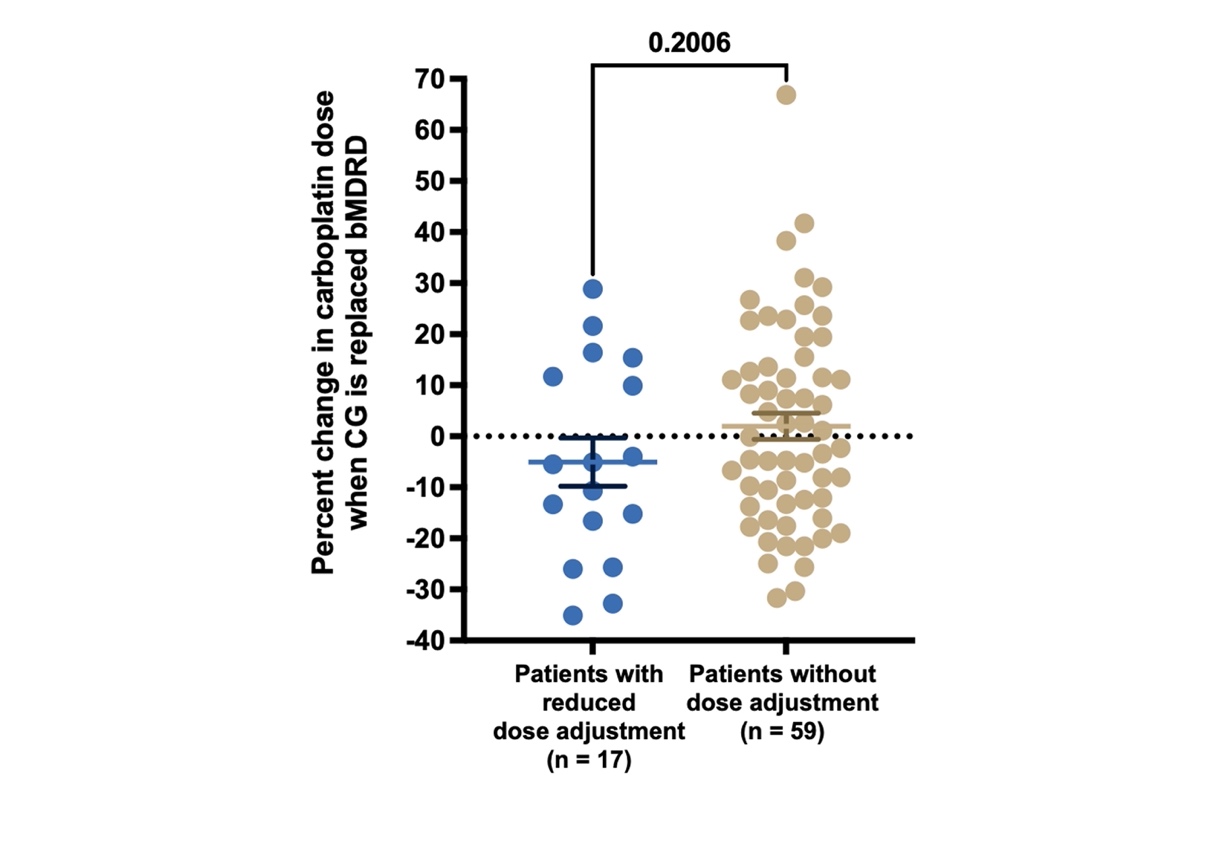


***Supplementary Fig. 4:*** *The patients who received a clinically reduced carboplatin dose were compared to those without dose adjustment in terms of their hypothetical carboplatin dose change as a result of substituting CG with MDRD. Data acquired from the 1^st^ cycle of carboplatin treatment.*


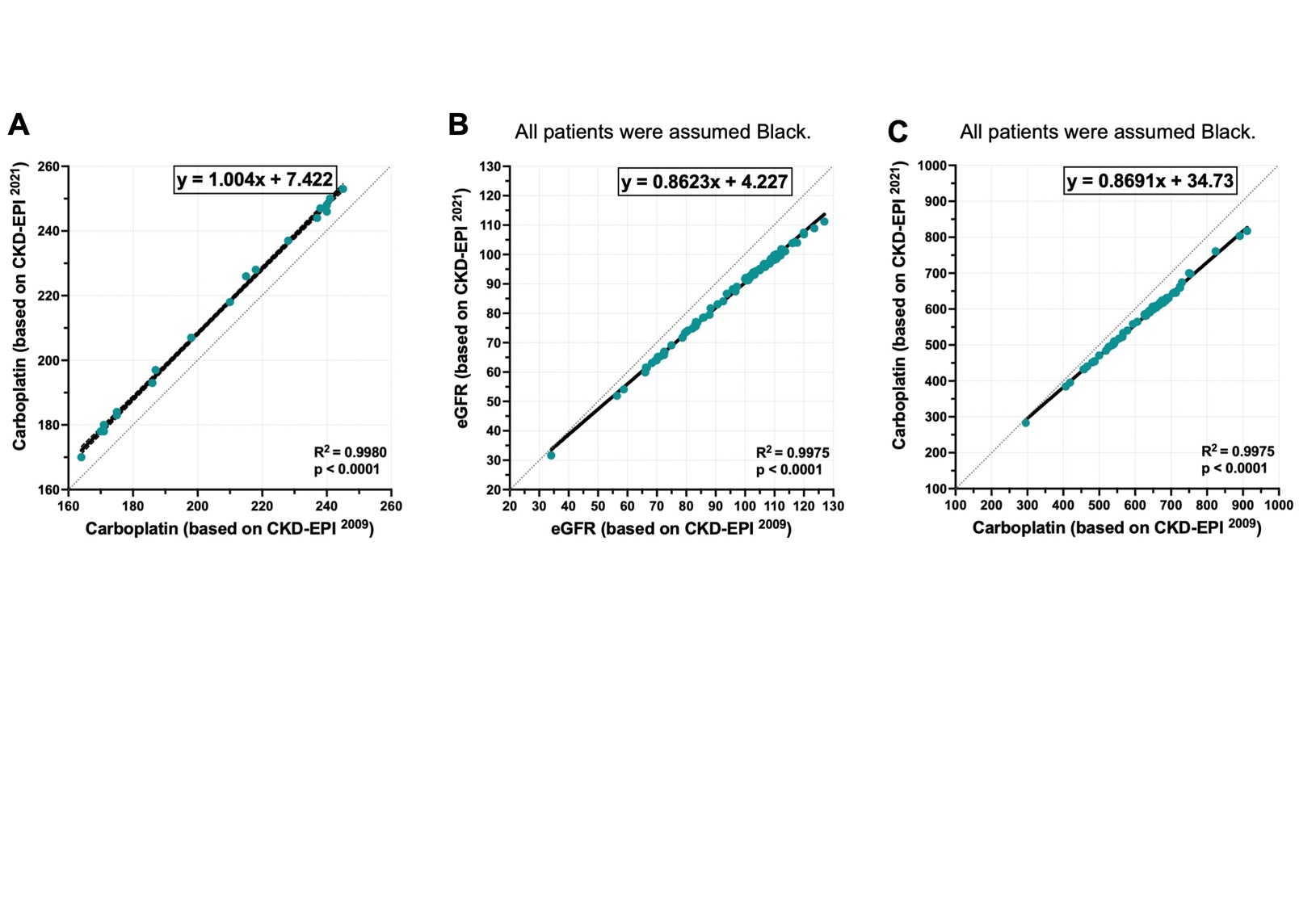


***Supplementary Fig. 5:*** *Simple linear regression models are used to describe the relationship between CKD-EPI ^2009^ (x-axes) and CKD-EPI ^2021^ (y-axes) equations.* ***A*** *The relationship based on carboplatin dosages* *using data from AUC-2 patients prior to the first carboplatin cycle (n = 19).* ***B and C*** *The impact of omitting race parameter in CKD-EPI ^2021^ equation is illustrated by charting the eGFR* ***(B)*** *and carboplatin* ***(C)*** *values after assuming that all patients were Black. The relationships are described by linear formulae, the p-values of the relationships and R^2^ values are indicated.*


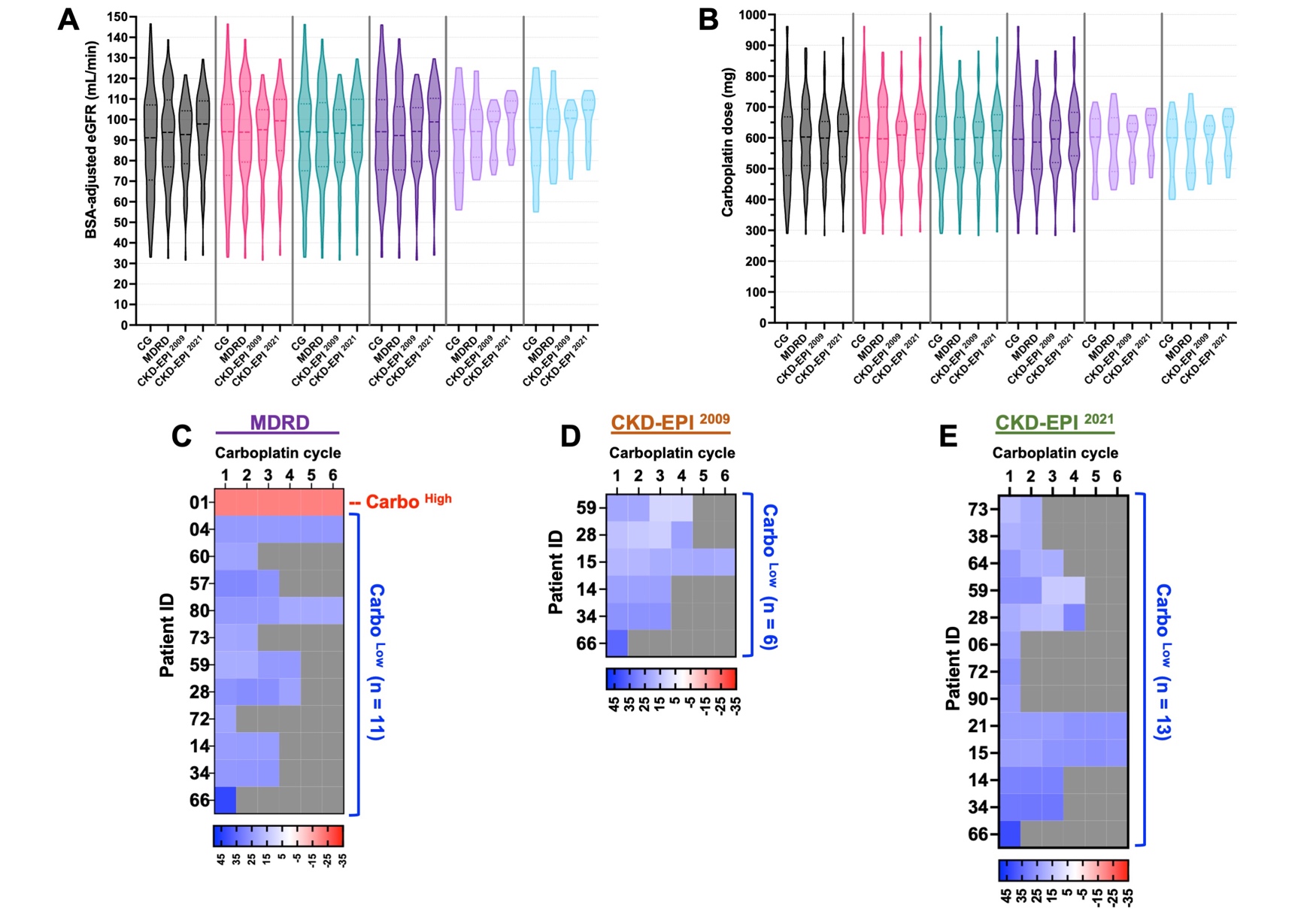


***Supplementary Fig. 6:*** *BSA adjustment has opposite impact on Carbo^High^ and Carbo^Low^ patient group size.* ***A-B,*** *Violin graphs showing the BSA-adjusted eGFR values* ***(A)*** *and BSA-adjusted carboplatin doses* ***(B)*** *calculated by CG, MDRD, CKD-EPI^2009^, and CKD-EPI^2021^ formulae. Values (eGFR or carboplatin dose) prior to each treatment cycle is indicated with a different colour. Median values and quartiles are shown with solid lines. Only AUC-5/6 patients are included.* ***C-E,*** *BSA-adjusted carboplatin changes for the patients who would have received a dose that is at least 20% different than their original dose in any of the six carboplatin cycles as a result of substituting CG with MDRD* ***(C)****, CKD-EPI ^2009^* ***(D)****, and CKD-EPI ^2021^* ***(E)****. Red boxes indicate BSA-Carbo ^High^ patients, and blue boxes indicate BSA-Carbo ^Low^ patients.*


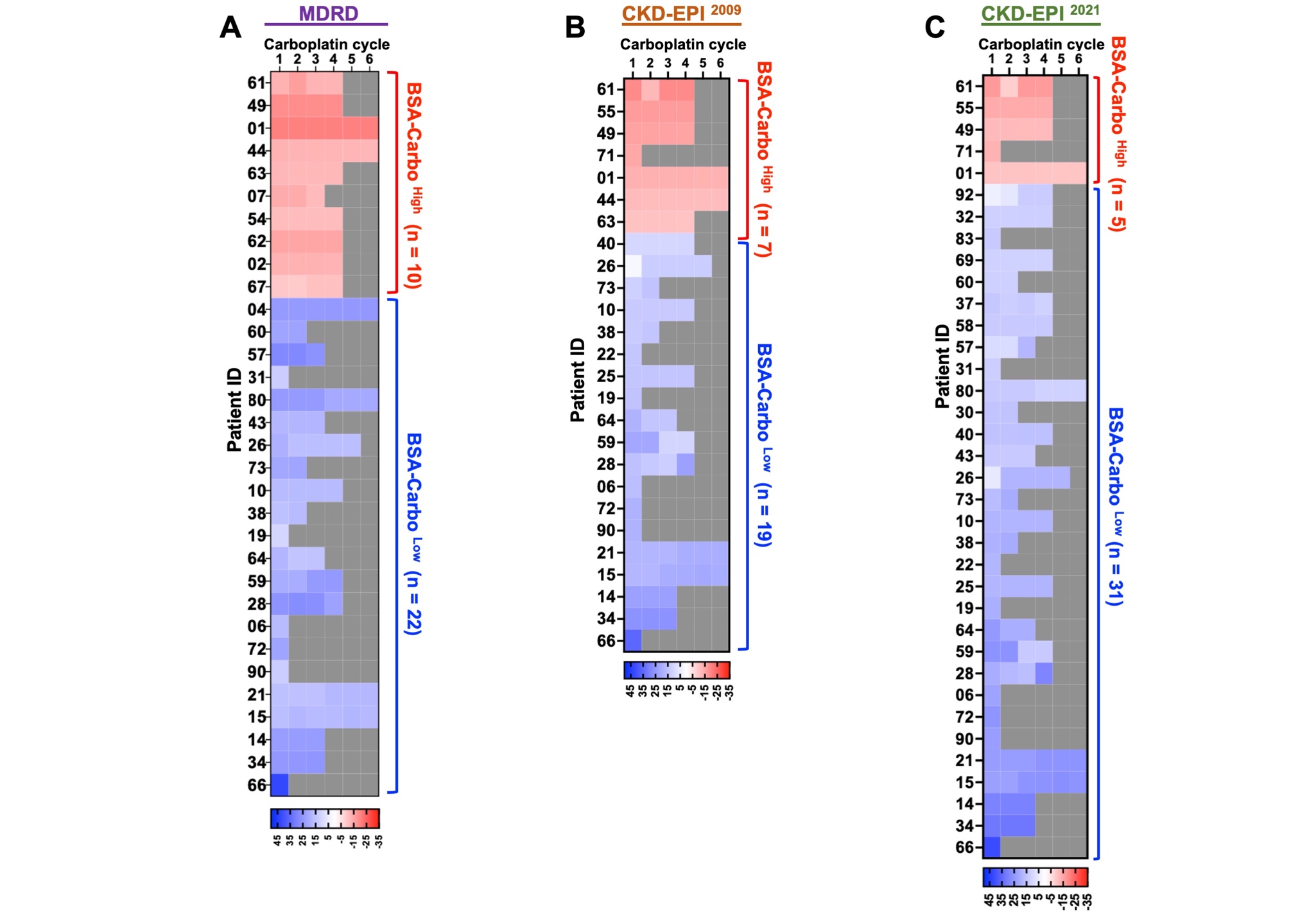


***Supplementary Fig. 7: A-C,*** *BSA-adjusted carboplatin dose changes for the patients who would have received a dose that is at least 10% different than their original dose as a result of substituting CG with MDRD* ***(A)****, CKD-EPI ^2009^* ***(B)****, and CKD-EPI ^2021^* ***(C)****. Red boxes indicate Carbo ^High^ patients, and blue boxes indicate Carbo ^Low^ patients.*


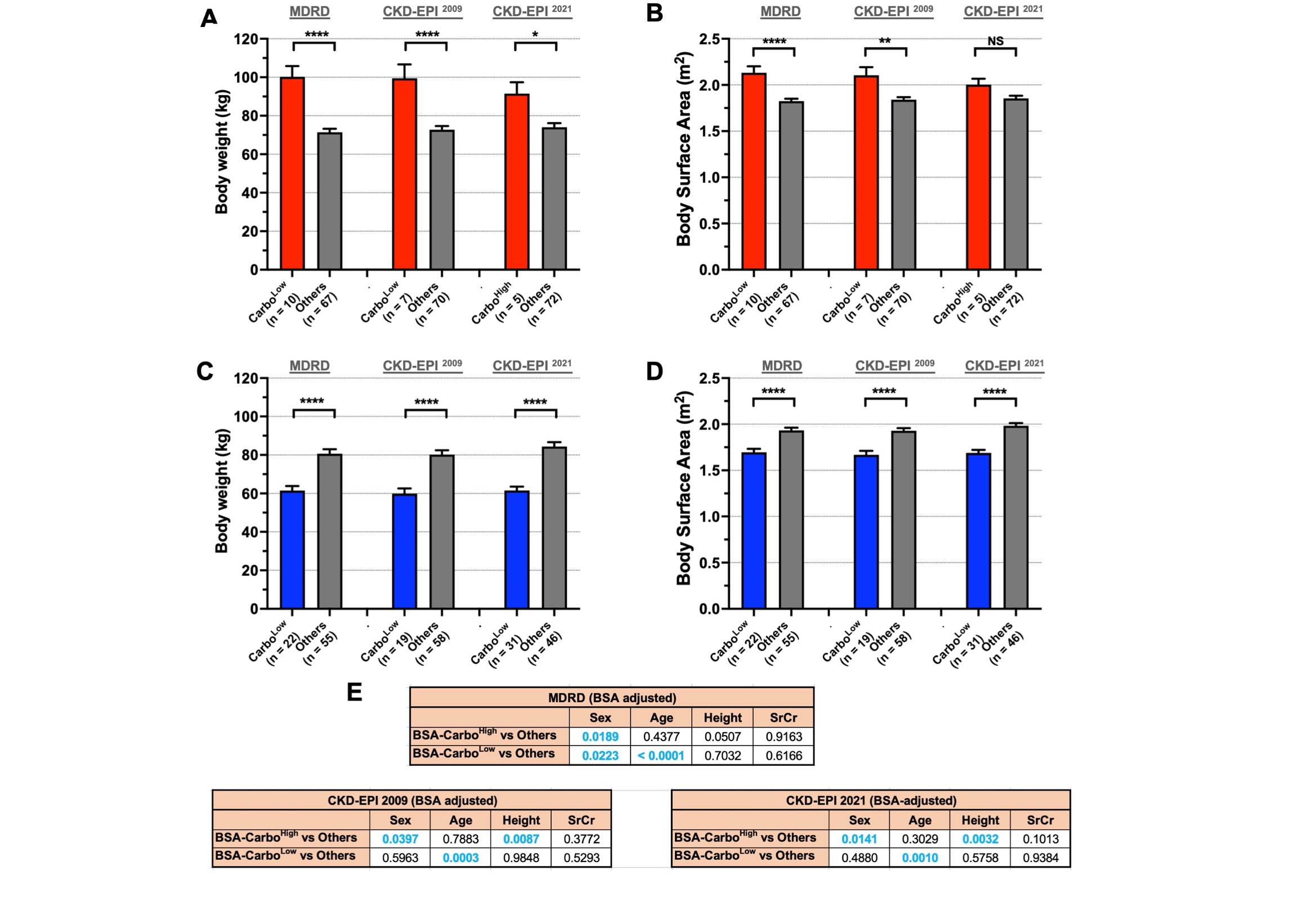


***Supplementary Fig. 8: A-D,*** *Patient characteristics (i.e., body weight and body surface area (BSA)) are compared between BSA-Carbo^High^ patients (red bars) and Others (grey bars)* ***(A and B)****, or BSA-Carbo^Low^ patients (blue bars) and Others (grey bars)* ***(C and D)*** *who were identified previously in Supp. Fig. 6. Body weight comparisons are shown in* ***A*** *and* ***C****; BSA comparisons are shown in* ***B*** *and* ***D****.* ***E,*** *Height is compared between BSA-Carbo^Low^ patients (blue bars) and Others (grey bars). The name of the formulae that have identified these patient groups are indicated at the top of the bars.* ***F,*** *Sex, age, height, and SrCr values are compared between BSA-Carbo^High^ patients and Others or BSA-Carbo^Low^ patients. The name of the formulae that have identified these patient groups are indicated at the top each table. Fisher’s exact test is used for sex comparison; unpaired, two-tailed t-test is used for all other statistical comparisons. Data from AUC-5/6 patients were used.*

**Supplementary Table (Raw Data.xlsx):** *The raw data regarding the Carboplatin treatment of the 96 patients with lung cancer was used in the current study, and listed in a table. This data set was acquired from the Treatment Chart (Pathways) and Drug Orders sections of the Charm software. The Treatment Chart data included the treatment pathway, cancer subtype, patient IDs, carboplatin cycle, date of data entry, dosing height, dosing weight, dosing body surface area (BSA), dosing eGFR, carboplatin date, AUC, carboplatin dose, adjusted dose. Data on Drug Orders included the date of data, height, weight (Wt) actual, BSA dosing, SrCr, GFR dosing. Each data point was collected up to 6 times depending on the number of carboplatin cycle number for each patient. Patients were deidentified using patient ID numbers (01-96).*
